# Metabolic Rewiring in MASLD: From Disease Mechanisms to Precision Medicine

**DOI:** 10.3390/metabo16080529

**Published:** 2026-07-27

**Authors:** Amedeo Lonardo, Ralf Weiskirchen

**Affiliations:** 1 Independent Researcher, 41100 Modena, Italy; 2 Institute of Molecular Pathobiochemistry, Experimental Gene Therapy and Clinical Chemistry (IFMPEGKC), RWTH University Hospital Aachen, D-52074 Aachen, Germany; rweiskirchen@ukaachen.de

**Keywords:** bile acids, biomarkers, cholesterol, gut–liver axis, lipidomics, MASH, mitochondria, phospholipids, saturated fatty acids, sex differences

## Abstract

**Highlights:**

**What are the main findings?**

- Development and progression of metabolic dysfunction-associated steatotic liver disease (MASLD) are characterized by staged metabolic rewiring.
- In this context, lipid overflow, impaired lipid disposal, mitochondrial dysfunction, oxidative stress, sterile inflammation, and fibrogenic remodeling drive the transition from healthy livers to steatosis first, and subsequently to metabolic dysfunction-associated steatohepatitis (MASH), fibrosis, cirrhosis, and hepatocellular carcinoma in a subset of cases.
- Metabolomic and lipidomic studies identify disease signatures, including enrichment of diacylglycerols, ceramides, cholesterol-related lipids, altered bile acids, amino-acid imbalance, gut-derived microbial metabolites, and depletion of protective polyunsaturated fatty acids, lysophosphatidylcholines, and phosphatidylcholines.

**What are the implications of the main findings?**

- Integrated metabolomics, lipidomics, and spatial multi-omics can support non-invasive diagnostic approaches, risk stratification, identification of at-risk MASH, therapeutic monitoring, and biologically informed patient subgrouping in line with precision hepatology approaches.
- Clinical translation will require standardized analytical workflows, transparent metabolite annotation, longitudinal multicenter validation, and integration with imaging, clinical scores, genetics, microbiome data, lifestyle habits, medications, sex, and ethnicity.

**Abstract:**

**Background/Objectives**: Metabolic dysfunction-associated steatotic liver disease (MASLD), a leading cause of chronic liver disease, encompasses a continuum from steatosis to metabolic dysfunction-associated steatohepatitis (MASH), fibrosis, cirrhosis, and hepatocellular carcinoma. This review aimed to synthesize current evidence on how metabolomic, lipidomic, and spatial multi-omic approaches illuminate MASLD pathogenesis and support precision hepatology. **Methods**: A structured narrative review was conducted through searches of PubMed, Scopus, and Web of Science, complemented by manual screening of key references. Studies were prioritized when they addressed MASLD biology, metabolic rewiring, lipid remodeling, mitochondrial dysfunction, inflammatory and fibrogenic pathways, gut–liver–adipose crosstalk, biomarker development, or therapeutic monitoring. **Results**: The reviewed evidence identifies MASLD as a systemic metabolic disorder shaped by excess lipid flux, enhanced de novo lipogenesis, impaired mitochondrial adaptation, oxidative and endoplasmic reticulum stress, sterile inflammation, and hepatic stellate-cell activation. Recurrent metabolomic signatures include altered amino acid, fatty acids, bile acid, and microbial co-metabolite pathways. Lipidomic studies consistently implicate depletion of protective polyunsaturated fatty acids, lysophosphatidylcholines, and phosphatidylcholines, in association with accumulation of diacylglycerols and ceramides, in the transition from steatosis to MASH and fibrosis. Emerging spatial and multi-omic analyses further resolve cell-specific metabolic niches involving hepatocytes, macrophages, endothelial cells, and stellate cells. **Conclusions**: Metabolomics provides a mechanistic and translational bridge between molecular injury, histological progression, and non-invasive risk stratification in MASLD. Future progress requires standardized analytical workflows, longitudinal validation, causal pathway interrogation, and integration with imaging, genetics, microbiome profiling, and treatment-response phenotyping. Clinical implementation will require standardized platforms, transparent metabolite identification, external validation across diverse populations, cost-effectiveness analyses, and regulatory-grade evidence of clinical utility.

## 1. Introduction

Steatotic liver disease (SLD) associated with systemic metabolic dysfunction now represents one of the dominant causes of chronic liver disease worldwide, encompassing a spectrum that extends from simple steatosis to steatohepatitis and advanced fibrosis, and whose nomenclature has evolved substantially over time [1,2]. Collectively, the principal nomenclatures used to define SLD, nonalcoholic fatty liver disease/nonalcoholic steatohepatitis, metabolic dysfunction-associated fatty liver disease/steatohepatitis and metabolic dysfunction-associated steatotic liver disease/steatohepatitis (NAFLD/NASH, MAFLD/MASH and MASLD/MASH, respectively), are not invariably and entirely interchangeable, as they differ in their diagnostic thresholds, inclusion and exclusion criteria, and prognostic associations [3,4]. However, NAFLD and MASLD identify largely overlapping populations with broadly similar clinical trajectories [5,6].

In 2002, Fiehn established the conceptual basis for metabolomics by framing metabolites as downstream products of cellular regulatory networks that integrate genetic variation and environmental perturbations. Thus, the metabolome provides a proximal functional readout of cellular state, linking genotype to phenotype and molecular mechanisms to clinical presentation [7]. Within the genome-to-phenotype hierarchy, metabolites provide the most proximal biochemical readout of cellular function. By integrating genetic, transcriptomic and proteomic regulation with environmental exposures, nutrient availability and disease-related stress, the metabolome captures functional perturbations that may not be evident from DNA, RNA or protein alone, bridging molecular mechanisms and clinical phenotype [8,9,10,11].

Because MASLD nomenclature is recent, foundational metabolomic and lipidomic studies from 2005–2022 remain relevant when earlier labels are interpreted under current criteria [12]. Metabolomics has reframed MASLD as a systemic metabolic disease and identified biomarkers for diagnosis, staging and therapy monitoring [13,14,15]. Convergent MASLD signatures include diagnostic panels, stage-specific lipid remodeling, lipotoxic mediators and gut–liver axis microbial metabolites. Multi-cohort analyses link MASLD with altered energy, amino acid, fatty acid and bile acid metabolism, while serum panels including guanidinoacetate and saccharopine outperform conventional non-invasive indices for clinically relevant fibrosis and inflammation [16]. Lipidomic studies further show progressive hepatic and circulating remodeling, with steatosis-to-MASH transition marked by loss of protective polyunsaturated fatty acids (PUFAs), lysophosphatidylcholines (LPCs), and phosphatidylcholines (PCs), and accumulation of DAGs and ceramides that promote lipotoxicity and insulin resistance [13,16,17,18,19].

However, metabolomic associations should not automatically be interpreted as causal. Many reported signatures derive from cross-sectional or observational studies and therefore identify metabolites associated with disease presence, stage, or prognosis rather than proven mechanistic drivers. Throughout this review, we distinguish disease-associated biomarkers from metabolites or lipid classes for which experimental, genetic, isotope-tracing, cellular, animal-model, or human tissue evidence supports a more direct mechanistic role in MASLD progression.

Unbalanced gut-derived metabolites also contribute to inflammation and fibrogenesis: increased levels of lipopolysaccharides/endotoxins, endogenous ethanol and acetaldehyde, Trimethylamine (TMA)/Trimethylamine N-oxide (TMAO), Hydrophobic secondary bile acids (e.g., deoxycholic acid) on the one hand and reduced microbial products, including indole propionic acid on the other hand, associate with greater fibrosis and ballooning [19,20,21]. Spatial metabolomics integrated with transcriptomics defines the metabolic microenvironment of advanced MASH, implicating macrophages and endothelial cells and enabling cellular- and spatially resolved mapping of metabolic reprogramming for targeted therapies [19,22,23,24].

Against this rapidly evolving background, the present review examines how metabolic rewiring drives the continuum of MASLD, from steatosis to MASH, fibrosis and cirrhosis. We integrate mechanistic insights into lipid overflow, mitochondrial dysfunction, oxidative stress, immune activation, gut–liver signaling and matrix remodeling with advances in metabolomics, lipidomics and spatial multi-omics. In doing so, we outline how molecularly defined metabolic states could refine risk stratification, non-invasive diagnosis, patient selection and mechanism-guided therapy in MASLD. We also discuss how recent spatial, single-cell, AI-assisted, and multimodal metabolomic approaches may strengthen this translational framework by linking circulating biomarkers to tissue-level mechanisms and clinically actionable prediction models.

## 2. Methods

The literature identification process followed a structured approach conceptually aligned with PRISMA principles to enhance transparency in this narrative review. Database searches were conducted in PubMed, Scopus, and Web of Science (WoS) using combinations of terms related to “MASLD,” “MAFLD,” “NAFLD,” “MASH,” “NASH,” “metabolomics,” “lipidomics,” “spatial metabolomics,” “multi-omics,” “biomarkers,” “fibrosis,” “mitochondrial dysfunction,” “lipotoxicity,” “bile acids,” “gut–liver axis,” “adipose tissue,” and “therapeutic monitoring.” Initial searches yielded a broad set of publications addressing MASLD, metabolomics, lipidomics, and mechanisms of metabolic liver disease. Duplicate records identified across databases were removed prior to screening. Titles and abstracts were screened to exclude studies not directly related to MASLD biology, metabolomics, lipidomics, or metabolic signaling pathways relevant to liver disease. Particular emphasis was placed on studies integrating metabolic profiling with clinical staging, histological assessment, fibrosis risk, biomarker development, therapeutic monitoring, or mechanistic investigation of lipid metabolism, mitochondrial dysfunction, inflammatory signaling, fibrogenesis, and gut–liver–adipose interactions. Full-text articles were subsequently evaluated for eligibility. Studies were retained if they reported metabolomic or lipidomic analyses, described metabolic pathways implicated in MASLD progression, or investigated circulating or tissue-derived metabolite signatures relevant to disease staging, fibrosis risk, or therapeutic monitoring. Experimental animal or cellular studies were included when they provided mechanistic insight into metabolic pathways that could help explain human disease phenotypes. Additional articles were identified through manual screening of reference lists from key reviews and high-impact original studies. This iterative process enabled the incorporation of emerging evidence related to spatial multi-omics, machine-learning approaches to metabolomic biomarkers, gut microbiota–derived metabolites, and treatment-response profiling in MASLD progression. Following full-text screening and relevance assessment, the final evidence base comprised clinical cohort studies, mechanistic experimental investigations, and multi-omics analyses considered most relevant to the scope and objectives of this narrative review.

## 3. Analytical Methodologies and Quality Control in MASLD Research

### 3.1. Analytical Methods for Identification of Metabolic Dysfunction-Associated Steatotic Liver Disease

Metabolomics research in MASLD relies on analytical platforms capable of capturing a chemically diverse spectrum of metabolites, ranging from polar intermediates of central carbon metabolism to highly hydrophobic lipid species [22]. The most widely used technologies include liquid chromatography–mass spectrometry (LC-MS), gas chromatography-mass spectrometry (GC-MS), and nuclear magnetic resonance spectroscopy (NMR), each providing complementary analytical strengths for metabolic profiling [13,22].

**Figure 1:**
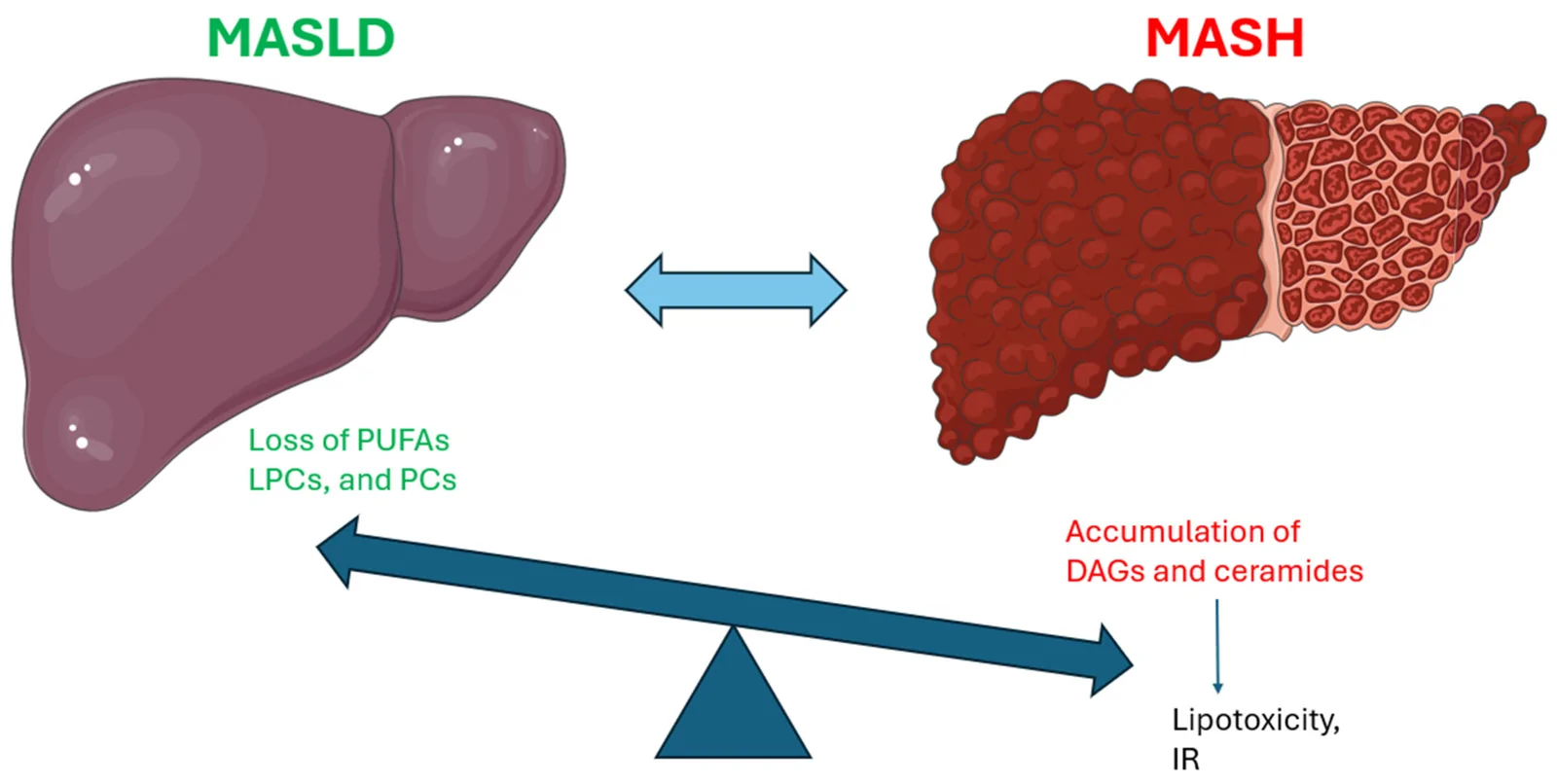
Lipidomic remodeling across metabolic dysfunction-associated steatotic liver disease (MASLD) progression. The figure summarizes the progressive hepatic and circulating lipid changes that accompany the transition from MASLD with simple steatosis to metabolic dysfunction-associated steatohepatitis (MASH). Early lipid overflow and enhanced de novo lipogenesis promote triglyceride storage, whereas advancing disease is characterized by depletion of protective polyunsaturated fatty acids (PUFAs), lysophosphatidylcholines (LPCs) and phosphatidylcholines (PCs), along with enrichment of diacylglycerols (DAGs), ceramides, and cholesterol-related lipid species. These lipidomic shifts favor lipotoxic stress, insulin resistance (IR), mitochondrial dysfunction, oxidative injury, and sterile inflammation, thereby linking metabolic overload to hepatocyte damage, fibrogenic remodeling and progression toward advanced liver disease. The specific metabolomic alterations associated with advanced fibrosis and cirrhosis are discussed under Section 5. The original illustration, based on references cited in the text, was created using Servier Medical ART (SMART) and is licensed under the Creative Commons Attribution 4.0 International License (CC BY 4.0).

LC-MS has become the dominant platform in MASLD metabolomics because of its sensitivity and broad coverage of lipid classes, bile acids, amino acids, and acylcarnitines. Coupled with high-resolution mass analyzers, LC-MS enables both targeted quantification of predefined metabolite panels and untargeted discovery of previously unrecognized metabolic perturbations. This approach has been particularly useful for identifying lipid remodeling signatures associated with the transition from steatosis to MASH, including accumulation of DAGs and ceramides and depletion of protective phospholipids and PUFAs (Figure 1).

GC-MS remains an essential complementary technique for profiling volatile or derivatized metabolites such as organic acids, short-chain fatty acids, and intermediates of the tricarboxylic acid cycle (TCA). Through chemical derivatization, GC-MS provides high chromatographic resolution and reproducible quantification of central carbon metabolites that reflect mitochondrial function and energy metabolism [25,26]. These features make it particularly useful for examining metabolic adaptations in early steatosis and mitochondrial dysfunction during disease progression.

NMR spectroscopy offers an orthogonal analytical approach characterized by high reproducibility and minimal sample preparation. Although its sensitivity is lower than that of mass spectrometry-based techniques, NMR provides quantitative and structurally robust information for abundant metabolites, including amino acids, glucose derivatives, and lipoprotein-associated lipid fractions [25,27]. This platform is therefore frequently used in large clinical cohorts to generate standardized metabolic fingerprints that can be correlated with disease stage and metabolic comorbidities.

Biological sample matrices strongly influence the type of metabolic information obtained. Liver tissue metabolomics derived from biopsy samples offers the most direct insight into hepatocellular metabolic pathways, allowing investigation of lipid droplet composition, mitochondrial metabolism, and intrahepatic signaling networks [13]. However, tissue sampling is invasive and therefore limited in clinical practice. Circulating matrices such as plasma or serum provide a minimally invasive alternative that captures systemic metabolic perturbations and enables biomarker discovery for non-invasive disease stratification [22]. Urine and fecal metabolomics further extend this framework by reflecting host–microbial co-metabolism and gut–liver axis activity [28,29].

Ensuring analytical reproducibility and metabolite identification confidence represents a central challenge in metabolomics [30]. Standardized quality control procedures typically include pooled reference samples, internal standards, batch randomization, and monitoring of instrument performance across analytical runs [30]. These approaches allow correction for signal drift, batch effects, and technical variability that could otherwise confound biological interpretation.

Metabolite identification is generally classified according to the Metabolomics Standards Initiative (MSI) framework, which defines four levels of identification confidence [31]. Level 1 corresponds to confirmed identification using authentic chemical standards and matched retention time and fragmentation spectra. Levels 2 and 3 represent putative annotation based on spectral similarity or molecular features, while Level 4 denotes unknown features detected but not structurally characterized [32]. Transparent reporting of MSI levels is essential to ensure reproducibility and comparability across studies.

In addition to analytical identification confidence, biological evidence levels should be considered when interpreting metabolomic signatures. In this review, we use the term “disease-associated biomarker” to denote a metabolite, lipid species, or metabolite pattern that is statistically associated with MASLD presence, disease stage, fibrosis risk, prognosis, or treatment response. We use “mechanistically supported metabolite” to denote a metabolite or lipid species for which experimental perturbation, genetic evidence, tissue localization, flux analysis, animal-model data, organoid studies, or pathway-specific evidence supports a functional role in disease biology. We reserve the term “causal mediator” for metabolites whose modulation has been shown to alter relevant MASLD phenotypes in experimental, longitudinal, genetic, or interventional frameworks.

To sum up, rigorous analytical workflows, standardized quality-control procedures, and transparent metabolite annotation frameworks are essential for translating metabolomics from discovery-based profiling toward clinically reliable biomarkers in MASLD research.

For biomarkers intended for clinical deployment, MSI level 1 confirmation should be prioritized whenever possible, because “misannotation” of structurally related lipids, bile acids, amino-acid derivatives, or microbial co-metabolites can compromise assay transferability, biological interpretation, and regulatory qualification.

Beyond analytical confidence, biological interpretation also requires caution. A metabolite that discriminates steatosis, MASH, or fibrosis in plasma or serum may function as a biomarker, a consequence of tissue injury, a compensatory response, or a true pathogenic mediator. Establishing causality generally requires additional evidence from experimental perturbation, stable-isotope tracing, flux analysis, genetic instruments, animal models, organoids, or spatially resolved human tissue studies. This distinction is particularly important for circulating metabolomic panels, which integrate signals from the liver, adipose tissue, intestine, skeletal muscle, kidney, immune system, diet, medications, and microbiome.

Table 1 summarizes the advantages, limitations, and clinical applicability of LC-MS, GC-MS, NMR, spatial metabolomics, and multi-omics platforms.

### 3.2. Emerging Spatial, Single-Cell, AI-Assisted, and Multimodal Metabolomics Approaches

Recent studies published in 2025–2026 have accelerated the transition from bulk metabolomic profiling toward spatially resolved, cell-aware, and computationally integrated approaches in MASLD research [15,23]. Spatially resolved multi-omics can map metabolic perturbations within liver tissue architecture, thereby linking lipid remodeling, bile-acid signaling, mitochondrial stress, and inflammatory mediators to hepatic zonation, fibrotic septa, inflammatory foci, and cellular neighborhoods. This is particularly relevant in MASH, where hepatocytes, macrophages, endothelial cells, and HSCs occupy distinct but interacting metabolic niches that may be obscured in bulk tissue extracts or circulating biofluids.

Single-cell and spatial single-cell omics further extend this concept by resolving cell-type-specific transcriptional and metabolic states during disease progression [23,33]. Although true single-cell metabolomics remains technically challenging because of low metabolite abundance, rapid metabolic turnover, ion suppression, limited coverage, and difficulties in metabolite annotation, integration of single-cell transcriptomics, spatial transcriptomics, imaging mass spectrometry, lipidomics, and computational pathway inference can approximate cell-specific metabolic programs. These approaches are particularly useful for identifying hepatocyte zonation defects, macrophage activation states, endothelial capillarization, and stellate-cell fibrogenic reprogramming in MASH.

AI-assisted metabolomic analysis and multimodal biomarker integration are also becoming increasingly important [34,35]. Machine-learning and deep-learning approaches can integrate high-dimensional metabolomic or lipidomic profiles with clinical chemistry, anthropometry, imaging, elastography, genetics, microbiome features, and spectroscopic data. Such models may improve classification of at-risk MASH, advanced fibrosis, or treatment response compared with single-modality biomarkers. However, their translational value depends on interpretability, calibration, external validation, locked analytical pipelines, and demonstration that model-guided decisions improve clinical outcomes.

These emerging approaches strengthen the translational perspective of metabolomics by moving the field from static circulating signatures toward spatially localized mechanisms, cell-type-resolved disease endotypes, and integrated clinical decision-support tools. At present, however, most remain research-stage technologies. Routine deployment will require standardized tissue handling, harmonized spatial resolution and annotation criteria, robust computational workflows, prospective validation, cost-effectiveness evidence, and regulatory qualification.

## 4. Stage-Specific Biochemical Disruptions and Metabolic Hallmarks of MASLD Progressions

### 4.1. The Steatosis Stage

SLD reflects an imbalance between lipid acquisition, disposal, and export [36]. Increased delivery of adipose-derived fatty acids, enhanced de novo lipogenesis (DNL), insufficient mitochondrial β-oxidation, and impaired triglyceride-rich lipoprotein secretion promote hepatic triglyceride retention and accumulation of bioactive lipid intermediates, notably DAGs and ceramides [37]. In insulin-resistant white adipose tissue, defective lipid-droplet regulation and excessive adipose triglyceride lipase (ATGL)- and hormone-sensitive lipase (HSL)-dependent lipolysis increase non-esterified fatty acid release, thereby linking adipose dysfunction to hepatic steatosis and lipotoxic stress [38].

Hepatic DNL is a central feature of insulin-resistant SLD. Chronic hyperinsulinemia and carbohydrate excess sustain SREBP-1c-dependent activation of the acetyl-CoA carboxylase–fatty acid synthase (ACC–FASN) axis, increasing palmitate generation and triglyceride deposition [38]. Re-esterification of exogenous and newly synthesized fatty acids through diacylglycerol acyltransferase (DGAT)1- and DGAT2-dependent pathways initially buffers free fatty acid toxicity within lipid droplets [37,38]. When this capacity is exceeded, lipid flux is redirected toward intermediates that impair insulin signaling, organelle function and hepatocyte viability [37].

DAG accumulation in hepatocytes and other insulin-sensitive tissues activates protein kinase C isoforms, including protein kinase C (PKC)ε in the liver and PKCθ in muscle, thereby impairing insulin receptor substrate-1 signaling and reinforcing insulin resistance [39,40].

### 4.2. Progression to Sterile, Dysmetabolic Steatohepatitis

Palmitate generated by DNL or delivered through adipose-derived lipid overflow can enter the de novo ceramide synthesis pathway through serine palmitoyltransferase. Ceramides disrupt Akt/PKB signaling, promote mitochondrial fragmentation and reactive oxygen species generation, and sensitize hepatocytes to apoptotic cell death [38,41].

Progression from steatosis to lipotoxic MASH reflects the convergence of cholesterol accumulation, oxidative stress, mitochondrial dysfunction, and inflammatory signaling. Together, these processes reshape hepatocellular metabolism and immune–stromal crosstalk, fostering pro-fibrogenic responses in susceptible individuals [42].

#### 4.2.1. Cholesterol

A study integrating human liver specimens with mechanistic evidence from mice fed a choline-deficient, L-amino acid-defined high-fat diet identified lipid-droplet cholesterol as a determinant of MASH severity [43]. Hepatic lipid-droplet cholesterol was enriched in individuals with MASH and fibrosis and was higher in carriers of the *PNPLA3* I148M variant (rs738409) than in those carrying protective hydroxysteroid 17-beta dehydrogenase 13 (*HSD17B13*) variants (rs72613567), supporting a genetic–metabolic link between cholesterol handling and fibro-inflammatory progression [43].

#### 4.2.2. Saturated Fatty Acids

Saturated fatty acids, particularly palmitate (C16:0), are prototypical lipotoxic species that promote cellular stress, mitochondrial dysfunction, and pro-inflammatory signaling [44]. Excess palmitate is diverted into sphingolipid and glycerolipid pathways, increasing ceramides and DAGs, which act as mediators of insulin resistance through PKC- and JNK-dependent stress signaling [45,46,47,48,49].

#### 4.2.3. Dysregulated Phospholipid Metabolism and Lipotoxic Stress

A pioneering study found that increased lipogenesis, desaturase, and lipoxygenase (LOX) activities characterize MASLD and MASH, whereas impaired peroxisomal PUFA metabolism and nonenzymatic oxidation are associated with progression to MASH [50]. In MASH, excess saturated free fatty acids (FFAs) and enhanced DNL disrupt phospholipid synthesis, lower the PC:PE ratio, and compromise membrane integrity. Saturated acyl chains rigidify endoplasmic reticulum (ER) membranes, activate the unfolded protein response, and promote chronic organelle stress. LPC accumulation further destabilizes plasma and mitochondrial membranes and triggers apoptosis through JNK activation and CHOP upregulation. Loss of membrane integrity releases damage-associated molecular patterns and lipotoxic extracellular vesicles that activate Kupffer cells and hepatic stellate cells (HSCs), driving sterile inflammation [44,51,52].

Transcriptomic studies identify increased expression of CD36 and regulatory non-coding RNAs, including Linc01271, as mechanisms that enhance hepatocellular uptake of circulating non-esterified fatty acids and amplify lipotoxic stress in MASLD and MASH [53,54,55].

#### 4.2.4. Mitochondrial Involvement

Mitochondrial dysfunction and altered TCA-cycle activity in MASLD and MASH have been reviewed elsewhere [56]. Briefly, early lipid excess may increase hepatic fatty-acid β-oxidation and TCA-cycle flux; with progression, this adaptive response can become inefficient, favoring energetic stress and inflammatory MASH [53]. Acetyl-CoA overload may further disturb zonated metabolism. Citrate can support DNL, whereas succinate may exit mitochondria and act as a pro-inflammatory signal [51,53,57,58,59].

High-resolution multi-omics studies indicate impaired oxidative phosphorylation, with reduced respiration through complexes I, II, and IV [59]. Disease progression is also associated with megamitochondria, loss of membrane potential, and disorganized or fragmented cristae [60]. Hepatocyte ballooning, a hallmark of MASH, has been linked to higher systemic and hepatic MDA levels [61], alongside visceral adiposity, insulin resistance, raised alanine aminotransferase (ALT), and hyperuricemia [62].

Mitophagy is a conserved quality-control pathway that removes damaged mitochondria and preserves cellular homeostasis [63]. By coordinating damage sensing, stress signaling, autophagosome recruitment, and lysosomal degradation, it helps maintain mitochondrial integrity in MASLD and liver fibrosis across hepatocytes, macrophages, HSCs, and liver sinusoidal endothelial cells (LSECs) [64].

In healthy mitochondria, PTEN-induced kinase 1 (PINK1) is rapidly degraded. After membrane depolarization, PINK1 accumulates on the outer mitochondrial membrane and phosphorylates ubiquitin and Parkin, promoting Parkin-dependent polyubiquitination and recruitment of autophagy receptors such as optineurin (OPTN) and p62. PINK1 or Parkin downregulation, or loss-of-function variation, can impair this amplification step and limit clearance of severely depolarized mitochondria [65].

Reduced expression of mitochondrial fusion and quality-control components, including mitofusin 1 (MFN1), mitofusin 2 (MFN2), optic atrophy 1 (OPA1) and the PINK1–Parkin axis, is consistent with impaired clearance of damaged mitochondria [60]. Membrane-compromised mitochondria can release oxidized mitochondrial DNA (mtDNA) into the cytosol or extracellular space, where it acts as a damage-associated molecular pattern [66]. Leaked mtDNA may activate cGAS-STING signaling, while persistent mitochondrial ROS can damage proteins and lipids, favor permeability-transition-pore opening, and contribute to apoptosis or regulated necrosis [67].

#### 4.2.5. Activation of Inflammatory Pathways

Lipotoxic stress, reactive oxygen species and damage-associated signals convert hepatic metabolic injury into a self-amplifying inflammatory program involving inflammasome activation, NF-κB/JNK signaling and recruitment of myeloid and lymphoid cells. Oxidized mtDNA released into cytosolic and extracellular compartments can engage TLR9 and activate cGAS–STING signaling [64]. In parallel, lipotoxic and oxidative stress converge on the NLRP3 inflammasome, promoting maturation of IL-1β and IL-18 [68].

TLR4 and ER stress pathways converge on NF-κB, driving transcription of canonical inflammatory mediators, including TNF-α, IL-6, IL-8 and MCP-1 (CCL2) [69].

Chemokine gradients promote recruitment of inflammatory Kupffer cells and monocyte-derived macrophages, neutrophils with NET-forming capacity, and cytotoxic CD8^+^ and Th17 lymphocytes [70,71]. These immune populations reinforce stellate-cell activation through profibrogenic mediators, including TGF-β1, thereby linking sterile inflammation to extracellular-matrix deposition and progressive fibrosis [72].

### 4.3. From Inflammation to Scarring: Mechanisms of Fibrogenesis in MASH

MASH fibrogenesis is a maladaptive wound-healing response driven by metabolic stress, immune-cell recruitment and HSC activation [73]. Long-standing clinical evidence indicates that baseline lobular inflammation is a key determinant of progression to advanced fibrosis in MASH, with inflammatory activity on the initial biopsy independently associated with subsequent fibrotic progression [74]. In 2016, a study by Ballestri et al. identified distinct independent predictors for liver fibrosis severity in NAFLD patients. These authors found that significant fibrosis was associated with waist circumference, metabolic syndrome, and ALT levels [62]. Advanced liver fibrosis was independently predicted by age, platelet count, insulin resistance, type 2 diabetes, and serum total cholesterol levels [62].

By transdifferentiating into extracellular matrix–secreting myofibroblasts, HSCs promote progressive scarring, increasing the risks of cirrhosis, hepatocellular carcinoma (HCC), and extrahepatic outcomes in MASLD [75]. Defining the crosstalk and molecular cascades that govern this process is essential for developing effective anti-fibrotic therapies [76].

#### 4.3.1. Cellular Drivers and Intercellular Crosstalk

In MASH, the fibrotic niche emerges from coordinated crosstalk among injured hepatocytes, resident Kupffer cells, recruited macrophages, HSCs and liver sinusoidal endothelial cells Zhou [73].

Accumulation of saturated fatty acids and free cholesterol induces ER stress, mitochondrial dysfunction and lytic hepatocyte death. Injured hepatocytes release damage-associated molecular patterns and reactive oxygen species into the space of Disse, generating local danger cues that initiate inflammatory and fibrogenic signaling [77,78,79].

HSCs are central effectors of MASH fibrosis, translating chronic metabolic and inflammatory stress into extracellular-matrix deposition [80]. Persistent injury drives quiescent, vitamin A–storing stellate cells towards proliferative, metabolically rewired myofibroblasts, marked by lipid-droplet loss, increased glycolysis, glutaminolysis and lactate production, and deposition of fibrillar collagens, mainly types I and III [81,82,83].

Metabolic stress also promotes liver sinusoidal endothelial cell capillarization, characterized by loss of fenestrations and reduced basement-membrane porosity, as described by DeLeve [84]. This dysfunctional endothelial state favors mesenchymal-cell activation and may contribute directly to sinusoidal extracellular-matrix deposition [85].

#### 4.3.2. Molecular Signaling Pathways

The conversion of quiescent HSCs into matrix-producing myofibroblasts is orchestrated by interconnected profibrogenic, mitogenic, metabolic and developmental signaling programs.

##### The TGF-β/Smad Axis

TGF-β1 is the dominant canonical driver of hepatic fibrogenesis. Engagement of its serine/threonine kinase receptors activates Smad2 and Smad3, which cooperate with Smad4 to induce transcription of fibrillar collagens, fibronectin and α-SMA [86].

##### The PDGF Pathway

PDGF provides the principal mitogenic signal for activated HSCs. Binding to PDGFR-β engages ERK and PI3K–Akt pathways [87], thereby sustaining myofibroblast proliferation, migration and survival [88]. Targeting the PDGF pathway may slow fibrotic progression of MASLD [89].

##### Metabolic and Epigenetic Reprogramming

HSC activation is accompanied by metabolic rewiring that supports matrix production, including increased glycolysis, glutaminolysis and succinate–GPR91 signaling. These metabolic inputs also reshape chromatin through histone modifications and DNA methylation, stabilizing the fibrogenic phenotype [90].

##### Emerging Developmental Cascades

Developmental pathways further reinforce the fibrotic niche. Multi-omic and functional studies implicate Hippo–YAP/TAZ and Hedgehog signaling, with intracellular free cholesterol activating TAZ in hepatocytes and YAP in HSCs to generate a feed-forward program that increases tissue stiffness and constrains fibrosis resolution [91].

Table 2 summarizes the principal pathophysiological axes implicated in the progression from MASLD to MASH, highlighting the interconnected roles of lipotoxicity, mitochondrial dysfunction, oxidative and organelle stress, inflammatory signaling and fibrogenic remodeling.

In conclusion, recent multi-omics studies delineate a coherent metabolic trajectory in which substrate excess, lipotoxic lipid species and progressive mitochondrial dysfunction converge to amplify oxidative stress, exemplified by increased malondialdehyde levels. This metabolic injury provides a permissive context for activation of the NLRP3 inflammasome and NF-κB-dependent inflammatory programs, thereby linking simple steatosis with the emergence of the progressive inflammatory, tissue-remodeling MASH variant.

### 4.4. Role of Gene Variants in Metabolic Rewiring in MASLD

MASLD reflects a genetically modulated failure to maintain hepatic lipid homeostasis under cardiometabolic stress. Rather than acting as isolated risk factors, common variants in *PNPLA3*, *TM6SF2*, *GCKR*, *MBOAT7* and *HSD17B13* define a coordinated pathogenic framework in which increased lipid input, impaired lipid-droplet turnover, defective lipoprotein export, altered membrane remodeling and modified injury-resolution pathways determine progression from steatosis to MASH, fibrosis and advanced liver disease [89].

Glucokinase regulator (*GCKR*) variation increases hepatic glucokinase activity and channels glycolytic carbon towards DNL, expanding the triglyceride pool available for storage [92,93]. In parallel, the *PNPLA3* I148M variant impairs lipid-droplet triglyceride remodeling and promotes retention of the mutant protein on lipid droplets, thereby limiting adaptive lipid mobilization. These complementary defects increase intracellular lipid burden and provide a metabolic substrate for lipotoxic stress [94,95,96,97].

Defective lipid disposal further amplifies this burden. *TM6SF2* E167K reduces secretion of triglyceride-rich VLDL particles, favoring hepatic triglyceride retention despite lower circulating lipoprotein concentrations. Consequently, hepatocytes increasingly rely on mitochondrial and peroxisomal fatty-acid oxidation to buffer lipid excess; when this compensatory capacity is exceeded, oxidative stress, endoplasmic-reticulum stress and inflammatory signaling promote the transition from metabolically buffered steatosis to MASH [97].

*MBOAT7* adds a distinct membrane-centered axis to this model. Reduced MBOAT7 activity alters phosphatidylinositol remodeling, changing the lipid composition of cellular membranes and sensitizing hepatocytes to organelle dysfunction, inflammatory activation and fibrogenic signaling [98,99,100]. By contrast, loss-of-function variation in *HSD17B13* attenuates liver injury and protects against chronic liver disease progression without necessarily preventing steatosis, highlighting the importance of downstream injury-resolution pathways as therapeutic targets [101,102,103].

Together, these loci support a unifying model of MASLD pathogenesis: inherited variation determines how the liver partitions, stores, exports and responds to lipid under metabolic pressure. Disease progression is therefore not simply a function of hepatic fat quantity, but of the balance between lipid flux, organelle resilience and the capacity to resolve cellular injury.

### 4.5. Role of Sex

Biological sex and reproductive status significantly influence the development and progression of MASLD. Sex chromosomes, gonadal hormones, adipose-tissue distribution, insulin sensitivity, gut-derived metabolites, and immune tone all contribute to sexually dimorphic liver metabolism. Reproductive milestones, including menarche, pregnancy, lactation, polycystic ovary syndrome, menopause, and exogenous hormone exposure, can further modify this background. Accordingly, the metabolomic profile of MASLD should be interpreted as the product of both biological sex and the changing endocrine milieu across the life course.

#### 4.5.1. Normal Liver to Simple Steatosis

In the transition from a metabolically healthy liver to simple steatosis, premenopausal women are generally less prone to hepatic fat accumulation than men, although this protection is relative and may be attenuated by obesity, insulin resistance, polyendocrine metabolic ovarian syndrome (PMOS), or adverse reproductive history [104]. Estrogen signaling favors subcutaneous rather than visceral fat storage, enhances insulin sensitivity, and supports hepatic fatty-acid oxidation, thereby limiting the intrahepatic overflow of saturated FFAs, DAGs, and ceramides [105]. Metabolomically, this profile is consistent with lower lipotoxic burden, more efficient handling of acylcarnitines and ketone bodies, and a comparatively preserved phosphatidylcholine-to-triacylglycerol balance.

Conversely, men more often display visceral adiposity, higher hepatic de novo lipogenesis, and greater exposure to lipotoxic lipid intermediates, favoring early accumulation of ceramides, DAGs, and long-chain acylcarnitines even before overt inflammatory injury [103]. However, the male pattern should not be viewed as uniformly deleterious nor the female pattern as uniformly protective.

Across sexes, several metabolomic alterations appear to accompany increasing severity of insulin resistance and metabolic SLD. In particular, higher circulating alanine, histidine, and tyrosine have been associated with more severe disease in both women and men [106]. Beyond these shared features, women with insulin resistance show a metabolic profile characterized by greater perturbation of glucose-related metabolites, triglyceride metabolism, glycoprotein acetyls (GlycA)—an inflammatory marker linked with early coronary heart disease risk—and saturated fatty-acid handling [107]. Consistent with this sex-specific susceptibility, a Finnish population study found that amino-acid profiles, including leucine, isoleucine, valine, and tyrosine, showed sex- and obesity-dependent interactions, with significant associations in women with abdominal obesity [108]. In women with NAFLD, glucose was positively associated with disease progression, whereas threonine showed an inverse association; changes in ketone bodies were also reported, including lower circulating acetoacetate and alterations in acetone, while the complex glycine response observed in female SLD warrants further study [106]. Men, by contrast, demonstrate higher baseline levels of hepatic lipogenic enzymes, leading to increased accumulation of ceramides and DAGs, which accelerates lipid deposition even under low-calorie metabolic stress [109]. Among male individuals, NAFLD progression was positively associated with higher serum valine, aspartic acid, and mannose, and inversely associated with creatine, phosphorylcholine and acetic acid, suggesting a more prominent amino-acid and phosphocholine-related signature in early disease evolution [106].

These differences suggest that simple steatosis may arise through partially overlapping but metabolomically distinct routes in males and females.

#### 4.5.2. Progression to MASH

During progression from simple steatosis to MASH, sex-related differences become more dependent on reproductive status. In postmenopausal women, declining estrogen concentrations and changes in body-fat distribution may shift the metabolomic pattern toward that observed in men, with higher levels of lipotoxic FFAs, ceramides, LPCs, oxidized lipid species, and incomplete fatty-acid oxidation products [110,111]. Yet convergence is incomplete. Men appear more likely to show early increases in BCAAs such as leucine, isoleucine, and valine, together with aromatic amino acids and insulin-resistance-associated metabolites, whereas women may exhibit more prominent disruption of bile-acid composition, choline and phosphatidylcholine metabolism, ketone-body availability, and one-carbon pathways [112]. These signatures can influence mitochondrial stress, endoplasmic-reticulum dysfunction, oxidative injury, and inflammasome activation, but their relative contribution may vary according to menopausal status, adiposity, diabetes, and endogenous hormone status and exposure.

#### 4.5.3. Transition to Fibrosis

The transition toward fibrosis is similarly shaped by sex and reproductive status, but the available metabolomic evidence remains less definitive than that for steatosis. In men, a faster fibrotic trajectory has been linked to persistent lipotoxicity, inflammatory sphingolipids, altered acylcarnitines, and metabolites that promote oxidative stress and HSC activation [111]. In premenopausal women, estrogen signaling may restrain HSC activation and extracellular matrix deposition, partly through anti-inflammatory and vasoprotective effects. After menopause, however, this restraint may weaken, and women may experience accelerated progression characterized by endothelial dysfunction markers such as ADMA, shifts in arginine and nitric-oxide metabolism, altered bile acids, and increased collagen-related amino-acid turnover, including proline- and hydroxyproline-associated pathways [113,114]. Importantly, these metabolomic changes are not exclusive to one sex; rather, they may differ in timing, intensity, and interaction with metabolic comorbidities.

**Figure 2:**
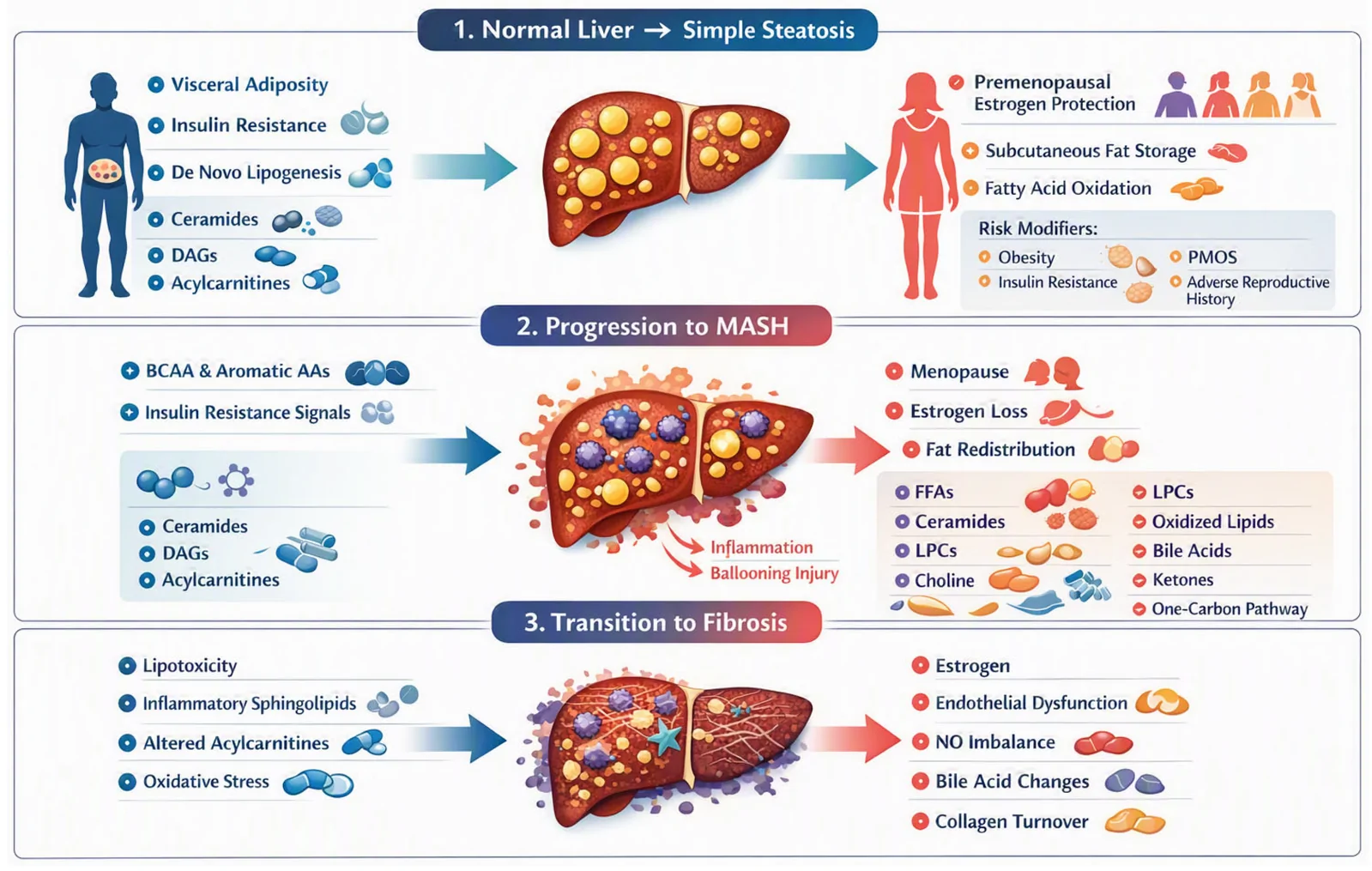
Sex-specific metabolomics in MASLD. Sex and reproductive status modulate lipid, bile-acid, amino-acid, and mitochondrial pathways, with estrogen-associated profiles linked to relative protection in premenopausal women and lipotoxic, pro-inflammatory, and pro-fibrogenic states more evident in men and postmenopausal women.

In summary, sex-specific metabolic variation appears to shape the trajectory from hepatic health to steatosis, MASH, and advanced fibrosis, while reproductive status determines when and how these differences emerge. A balanced interpretation is that estrogen-associated lipid, bile-acid, amino-acid, and mitochondrial signatures may contribute to the relative protection observed in many premenopausal women, whereas men and postmenopausal women more commonly converge on lipotoxic, pro-inflammatory, and pro-fibrogenic metabolic states. Future metabolomic studies should therefore stratify participants not only by sex, but also by menopausal status, PMOS, pregnancy history, lactation, hormone therapy, and cardiometabolic comorbidities, to avoid masking clinically relevant pathways and to support precision risk stratification in MASLD and MASH. Figure 2 summarizes the principal features of sex-specific metabolomics in MASLD.

## 5. Metabolomics of Advanced Fibrosis and Cirrhosis

**Figure 3:**
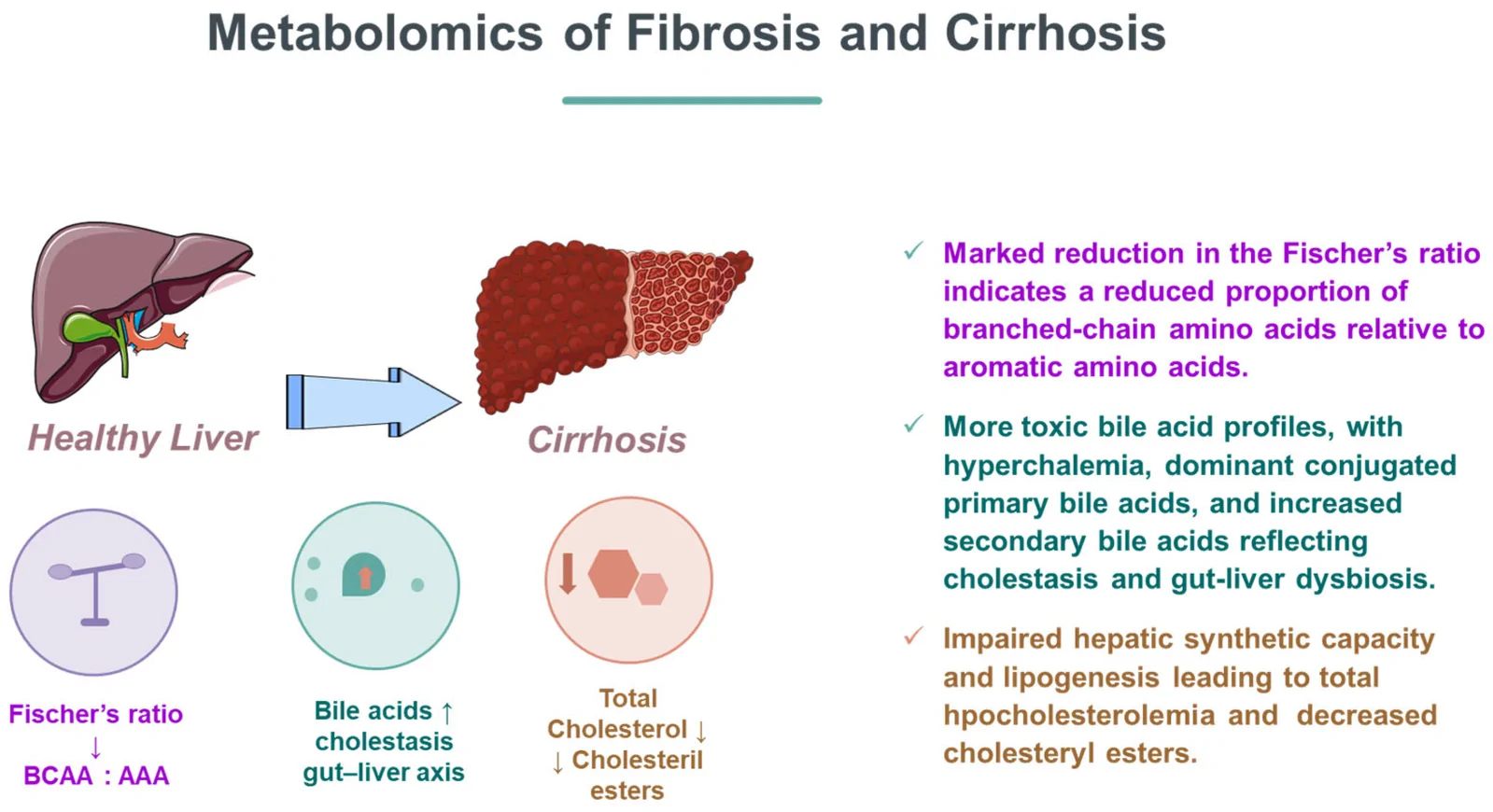
Metabolomics of advanced fibrosis and cirrhosis. Schematic overview of the major metabolomic alterations associated with advanced fibrosis and cirrhosis. Progressive liver dysfunction is characterized by a decline in the ratio of branched-chain to aromatic amino acids, reflecting impaired hepatic amino acid handling and increased extrahepatic branched-chain amino acid utilization. In parallel, circulating bile acid pools expand, with enrichment of conjugated primary and secondary bile acids, consistent with cholestasis and altered gut–liver axis metabolism. Loss of hepatic synthetic and lipogenic capacity further results in hypocholesterolemia and reduced circulating cholesteryl ester concentrations. The original illustration, based on references cited in the text, was created using Servier Medical ART (SMART) and is licensed under the Creative Commons Attribution 4.0 International License (CC BY 4.0).

The progression of chronic liver injury to advanced fibrosis and cirrhosis is driven by profound systemic metabolic rewiring, resulting in a distinct, multi-class metabolomic signature (Figure 3).

A hallmark feature of this transition is alteration of the amino acid profile, primarily characterized by a marked decline in Fischer’s ratio (i.e., the proportion of branched-chain amino acids to aromatic amino acids). This occurs as branched-chain amino acids (BCAAs, such as leucine, isoleucine, and valine) are preferentially consumed by skeletal muscle, while aromatic amino acids (such as phenylalanine and tyrosine) accumulate in circulation due to impaired hepatic clearance [115]. Concurrently, bile acid metabolism shifts toward a highly toxic profile; advanced fibrosis correlates with marked elevations in total serum bile acids. This increase is specifically dominated by conjugated primary bile acids, such as glycocholic acid and taurochenodeoxycholic acid, alongside secondary bile acids like deoxycholic acid (DCA), reflecting both progressive cholestasis and gut–liver axis dysbiosis [116,117,118,119].

Furthermore, as functional liver architecture collapses, disrupted hepatic synthetic capacity severely alters lipogenesis, leading to a progressive reduction in circulating total cholesterol and cholesteryl esters [120,121].

Together, these coordinated fluctuations in amino acids, bile acids, and cholesterol form a highly sensitive, non-invasive metabolomic framework capable of tracking tissue remodeling, evaluating advanced staging, and predicting clinical complications in end-stage liver disease [122].

## 6. Mechanistic Sub-Pathways (The Gut–Liver–Adipose Axis)

MASLD is increasingly recognized as a systemic metabolic disorder in which metabolic signals originating from the intestine, adipose tissue, and liver interact through a dynamic network of nutrient flux, microbial metabolites, and inflammatory mediators [123]. The gut–liver–adipose axis represents a central pathophysiological framework linking dietary inputs, microbial metabolism, adipose tissue dysfunction, and hepatic metabolic stress [124].

### 6.1. Microbial Co-Metabolites

The intestinal microbiota contributes significantly to host metabolic homeostasis by transforming dietary substrates into bioactive signaling molecules that influence hepatic metabolism and systemic inflammation. Among the most extensively studied microbial metabolites are short-chain fatty acids (SCFAs), TMAO, and secondary bile acids [125]. Alterations in the production or systemic distribution of these molecules have been increasingly associated with MASLD progression and fibrogenesis. SCFAs such as acetate, propionate, and butyrate are produced by bacterial fermentation of dietary fibers in the colon. These metabolites regulate host energy metabolism through multiple mechanisms, including activation of G-protein-coupled receptors (GPCRs), modulation of intestinal barrier integrity, and regulation of hepatic gluconeogenesis and lipid metabolism [126]. Reduced SCFA production has been reported in dysbiotic gut microbiota associated with MASLD, potentially contributing to increased intestinal permeability, endotoxin translocation, and hepatic inflammation. Another major microbial metabolite implicated in cardiometabolic disease is TMAO [126]. Dietary nutrients such as choline, PC, and L-carnitine are metabolized by intestinal bacteria to trimethylamine (TMA), which is subsequently oxidized in the liver by flavin-containing monooxygenases to generate TMAO. Elevated circulating TMAO levels have been linked to insulin resistance, inflammation, and lipid metabolic disturbances, suggesting that dysregulated microbial choline metabolism may contribute to hepatic steatosis and cardiovascular complications in MASLD [126].

Gut microbial dysbiosis may also generate harmful metabolites that promote inflammation and fibrogenesis. Increased production of lipopolysaccharides and endogenous ethanol, associated with reduced levels of protective metabolites such as indole derivatives, has been associated with increased hepatic inflammation and fibrosis severity in MASLD [127]. These findings support the concept that microbial–host co-metabolism contributes significantly to the inflammatory microenvironment driving MASH progression.

Bile acid metabolism provides another critical interface between the gut microbiome and hepatic metabolic regulation [128,129]. Primary bile acids synthesized in hepatocytes are released into the intestine, where microbial enzymes convert them into secondary bile acids such as deoxycholic acid. These molecules function not only as detergents facilitating lipid absorption but also as signaling ligands for nuclear receptors such as the Farnesoid X receptor (FXR) and the membrane receptor TGR5 [130]. Dysregulated bile acid signaling in MASLD can alter hepatic lipid metabolism, glucose homeostasis, and inflammatory responses, thereby reinforcing metabolic liver injury.

### 6.2. Adipose Tissue Crosstalk

Adipose tissue represents another major metabolic organ influencing MASLD development through systemic lipid flux and inflammatory signaling [131,132]. In conditions of obesity and insulin resistance, adipose tissue undergoes profound metabolic and inflammatory remodeling characterized by adipocyte hypertrophy, immune-cell infiltration, and dysregulated lipolysis [132]. These changes result in excessive release of non-esterified FFAs into the circulation. Because visceral adipose tissue drains directly into the portal circulation, FFAs released during enhanced lipolysis are delivered efficiently to the liver. The resulting increase in hepatic fatty acid influx contributes substantially to triglyceride accumulation and lipid droplet expansion within hepatocytes. When the capacity for safe triglyceride storage or mitochondrial oxidation is exceeded, excess fatty acids are redirected toward lipotoxic pathways that generate DAGs, ceramides, and other bioactive lipid intermediates [132].

Adipose tissue inflammation further amplifies this process. Pro-inflammatory cytokines such as tumor necrosis factor-α and interleukin-6 released from adipose tissue macrophages promote systemic insulin resistance and enhance hepatic DNL. At the same time, adipokines including adiponectin, leptin, and resistin exert complex regulatory effects on hepatic lipid metabolism and fibrogenesis [133]. Reduced adiponectin levels, frequently observed in obesity and MASLD, impair fatty acid oxidation and anti-inflammatory signaling in hepatocytes. This bidirectional communication between adipose tissue and liver therefore establishes a metabolic feedback loop in which adipose dysfunction drives hepatic lipid accumulation and inflammation, while hepatic metabolic stress further exacerbates systemic insulin resistance and adipose inflammation. Integration of these signals with microbial metabolites from the gut creates a complex metabolic network that shapes MASLD progression and may represent a target for therapeutic intervention.

## 7. Biomarker Panels and Precision Medicine Applications

Metabolomics has a dual role in advancing precision medicine for MASLD. First, metabolite-based signatures may improve diagnostic, translational, and prognostic stratification, thereby reducing reliance on liver histology for disease staging and risk assessment. Second, longitudinal metabolic profiling offers a non-invasive approach to monitoring treatment response and capturing pathway-level effects of lifestyle, pharmacological, or mechanism-targeted interventions.

Published multi-metabolite diagnostic models are summarized in Table 3, which illustrates their performance relative to established non-invasive tools, including FIB-4 and transient elastography.

Metabolomic profiling is moving from biomarker discovery toward pragmatic clinical use in MASLD and MASH. Several blood-based or serum-based panels now show clinically relevant performance for identifying advanced fibrosis, at-risk MASH, or histological MASH, particularly when combined with routine variables such as BMI, AST, and ALT [134,137,142,144,145]. This trajectory is clinically important because everyday MASLD care depends less on detecting steatosis than on identifying the smaller subgroup with progressive fibro-inflammatory disease who require specialist referrals, closer monitoring, or treatment consideration.

Recent AI-assisted and multimodal approaches further extend this translational trajectory. Machine-learning models using combined clinical and metabolomic features have achieved promising performance for identifying histological MASH and predicting liver-related outcomes, while multimodal deep-learning frameworks integrating spectroscopic data, clinical-biochemical variables, and bile-acid metabolomics illustrate how complementary data streams may be combined for non-invasive MASH diagnosis [34,35]. These approaches are attractive because they can capture nonlinear relationships among metabolic pathways, routine laboratory markers, and imaging- or spectroscopy-derived phenotypes. However, they should be interpreted as decision-support tools rather than autonomous diagnostic systems until externally validated, calibrated across populations, and tested prospectively within clinical pathways.

In practical terms, metabolomic tools are already available in some clinical and commercial settings, but they are not yet embedded as first-line tests in most MASLD pathways. Current care algorithms still prioritize inexpensive, widely accessible tests such as FIB-4, followed by elastography or established proprietary blood tests for patients with indeterminate or high-risk results. Metabolomic panels are therefore best viewed at present as second-line or adjunctive tools: they may help refine risk in patients with equivocal conventional non-invasive tests, support triage when elastography is unavailable or difficult to access and enrich clinical trial populations for patients with at-risk MASH.

The clearest near-term use case is risk stratification rather than population screening. Panels such as MASEF and related metabolome-derived scores are designed to identify patients with clinically consequential disease rather than to replace simple primary-care screening tools [136,138]. Their value may be greatest in patients with type 2 diabetes, obesity, metabolic syndrome, unexplained aminotransferase elevation, or imaging-detected steatosis, especially when standard scores and liver stiffness measurements yield discordant or borderline results. In these scenarios, metabolomics could reduce unnecessary liver biopsy and improve referral precision.

The clinical utility of metabolomics also depends on the question being asked. A signature optimized for advanced fibrosis is not automatically suitable for diagnosing active steatohepatitis, predicting HCC, or monitoring therapeutic response. This distinction is important because the studies summarized in Table 2 address different endpoints, biological matrices, and levels of evidence: some evaluate patient-facing diagnostic models [134,137,142,144,145], whereas others explore mechanisms, gut–liver biology, oncogenic progression, or treatment-related metabolic shifts [136,138,139,140,141,143]. Translation therefore requires each panel to be validated for a defined clinical purpose.

For everyday practice, a clinically useful metabolomic test would need to add information beyond FIB-4, elastography, and conventional laboratory panels, provide a clear rule-in or rule-out threshold, and return results quickly enough to influence referral, treatment or follow-up decisions. It should also be interpretable for non-specialists, reproducible across laboratories and populations, and cost-effective at the health-system scale. These requirements are particularly relevant as pharmacological treatment for non-cirrhotic MASH with significant fibrosis expands the need for accurate identification of eligible patients.

Several limitations still prevent widespread implementation. Many published models derive from tertiary-care or biopsy-enriched cohorts, which may overestimate performance in primary care or community populations. Analytical platforms differ across studies, pre-analytical variables are incompletely harmonized, and metabolite concentrations are influenced by diet, medications, fasting status, diabetes, renal function, alcohol exposure, genetics, sex, and ethnicity. Machine-learning models also require rigorous calibration, external validation, and transparent reporting before they can be trusted for individual patient decisions [137,144,145].

A further interpretative limitation is that metabolomic scores developed for risk stratification are not necessarily mechanistic models of MASLD pathogenesis. In many panels, metabolites are selected because they improve classification performance, not because each metabolite has proven causal relevance. Therefore, diagnostic or prognostic signatures should be interpreted primarily as disease-associated biomarker tools unless individual components are supported by experimental, genetic, or tissue-based mechanistic evidence. This distinction is especially important for machine-learning-derived scores, in which predictive variables may capture correlated systemic metabolic states rather than liver-specific causal pathways.

These translational barriers are particularly relevant for spatial, single-cell, AI-assisted, and multimodal metabolomic approaches, because these methods introduce additional layers of analytical complexity, computational integration, and clinical interpretability. They can be grouped into analytical, computational, clinical, economic, and regulatory domains, each requiring specific actions before metabolomic biomarkers can be implemented in routine MASLD care (Table 4).

The barriers summarized in Table 4 indicate that clinical implementation of metabolomics depends not only on diagnostic accuracy, but also on analytical reproducibility, transparent metabolite identification, robust external validation, interpretability, affordability, workflow integration, and regulatory readiness. These requirements are especially important for spatially resolved and AI-assisted tools, whose apparent performance may be inflated by pre-analytical tissue effects, site-specific bias, overfitting, data leakage, or insufficient calibration.

Availability is another practical constraint. Even when targeted metabolomic assays can be performed in certified laboratories, access, reimbursement, turnaround time, and integration into electronic clinical workflows vary substantially. In addition, clinicians need evidence showing that acting on metabolomic results improves patient outcomes, not merely diagnostic discrimination. Prospective studies should therefore test metabolomic panels within stepwise pathways, comparing them directly with elastography, imaging, and established blood tests while measuring referral efficiency, biopsy avoidance, treatment eligibility, longitudinal outcomes, and cost-effectiveness.

Metabolomics may ultimately be most powerful when used dynamically. Longitudinal profiling could capture pathway-level responses to weight loss, cardiometabolic optimization, or mechanism-targeted therapies, and experimental studies already suggest that treatment-related metabolic shifts can be measured [141,143]. However, human intervention studies are needed to determine whether changes in metabolomic signatures track histological improvement, fibrosis regression, liver-related outcomes, or treatment response sufficiently well to guide clinical decisions.

Thus, the translational message is balanced. Metabolomic profiling is no longer purely exploratory: selected assays and derived scores are already close to clinical deployment and may be useful in specialist pathways, clinical trials, and selected high-risk patients. Nevertheless, routine use across primary care and hepatology will require standardized assays, population-specific thresholds, independent validation, clear clinical algorithms, reimbursement models, and proof that test-guided management improves outcomes.

External validation remains essential because many existing models and applied techniques were derived from tertiary-care, biopsy-enriched, geographically or sex-restricted, or single-platform cohorts and may overestimate performance in primary-care or community settings [146,147,148]. Future studies should validate locked metabolomic and multimodal algorithms across ancestries, ethnicities, sex and reproductive-status groups, age ranges, obesity phenotypes, diabetes status, medication exposure, dietary patterns, renal function, alcohol intake, and microbiome backgrounds. Such studies should also determine whether metabolomics-guided pathways provide added value over FIB-4, elastography, imaging, and established blood tests, while meeting requirements for affordability, clinically useful turnaround times, workflow integration, regulatory qualification, and post-implementation performance monitoring.

In conclusion, metabolomics should currently be regarded as an emerging adjunct to, rather than a replacement for, established non-invasive MASLD risk-stratification pathways. Its most immediate role is to improve precision in patients at increased metabolic risk or with indeterminate conventional testing, while its longer-term promise lies in treatment monitoring and biologically informed patient stratification. Realizing this promise will require moving from statistically promising signatures to standardized, interpretable, externally validated, cost-effective, reimbursable, and regulatory-ready tools that clinicians can use confidently in everyday practice.

## 8. Current Technical Bottlenecks, Limitations and Future Outlook

Despite major advances in metabolomics and lipidomics technologies, several methodological and conceptual challenges continue to limit the translation of metabolic signatures into clinically actionable biomarkers for MASLD. Addressing these bottlenecks will be essential for moving the field from exploratory profiling toward standardized diagnostic and prognostic tools. One major challenge concerns reproducibility across many metabolomics studies [149].

From a clinical implementation perspective, these reproducibility issues are not merely technical details but prerequisites for regulatory-grade biomarker development. Inter-platform variability can arise from differences in chromatographic separation, ionization mode, mass analyzer type, NMR acquisition parameters, spectral libraries, peak detection, feature alignment, normalization, and metabolite annotation. There are established roadmaps for best practices within quality assurance and quality control practices [150]. Batch effects may reflect instrument drift, reagent lots, operator differences, sample order, matrix effects, and long-term storage conditions. Clinical translation therefore requires standardized analytical pipelines with prespecified quality-control metrics, pooled reference materials, internal standards, calibration procedures, batch-randomization strategies, drift correction, and transparent reporting of missing-data handling and statistical modeling [151]. Without such harmonization, the same biological sample may generate different metabolomic signatures across laboratories, limiting reproducibility and regulatory acceptance.

Differences in sample preparation, analytical platforms, chromatographic methods, and computational pipelines can produce substantial variability in detected metabolite profiles. Even when similar analytical technologies are used, variations in data preprocessing, peak alignment, normalization strategies, and metabolite annotation algorithms may lead to divergent results across research groups [149]. These methodological discrepancies contribute to the so-called reproducibility crisis in metabolomics, in which metabolic signatures identified in discovery cohorts are not consistently replicated in independent studies.

Biological heterogeneity within clinical cohorts further complicates interpretation of metabolomic data. Dietary patterns, medication exposure, ethnicity, microbiome composition, and metabolic comorbidities such as diabetes or obesity can all influence circulating metabolite profiles [152]. Without careful cohort characterization and statistical adjustment, these phenotypic heterogeneities may confound associations between specific metabolites and disease stage [153]. For example, lipidomic signatures observed in MASLD patients may partially reflect differences in diet, lipid-lowering medications, or genetic background rather than disease-specific metabolic pathways. Another emerging challenge involves the integration of metabolomics with other layers of biological information [154]. Metabolites represent downstream products of complex regulatory networks involving gene expression, protein activity, and environmental influences. As a result, metabolomic data alone may not fully explain the upstream mechanisms responsible for observed metabolic perturbations. Integrating metabolomics with transcriptomics, proteomics, lipidomics, and spatial multi-omics technologies offers a promising strategy to overcome this limitation. Such multi-layer approaches can reveal coordinated regulatory networks linking genetic variation, signaling pathways, and metabolic phenotypes in MASLD.

Recent studies combining spatial transcriptomics and metabolomics have demonstrated how metabolic rewiring varies across different cellular niches within the liver, highlighting the roles of hepatocytes, macrophages, endothelial cells, and stellate cells in shaping the metabolic microenvironment of MASH [33,155]. By mapping metabolic activity within tissue architecture, these technologies provide mechanistic insight that cannot be obtained from bulk metabolomic measurements alone. Open-science practices are also becoming increasingly important for ensuring transparency and reproducibility in metabolomics research. Public repositories such as MetaboLights and the Metabolomics Workbench now allow investigators to deposit raw spectral data, experimental metadata, and metabolite annotation files [156]. These platforms enable independent validation, cross-study comparison, and reanalysis of datasets using updated computational pipelines. Increasingly, journals and funding agencies require deposition of metabolomics datasets in public repositories as a prerequisite for publication, reflecting the growing emphasis on data transparency in systems biology research.

Several limitations should also be acknowledged when interpreting current metabolomics findings in MASLD. First, many studies rely on cross-sectional cohort designs, which limit the ability to determine whether observed metabolic alterations represent causal drivers of disease progression or downstream consequences of hepatic injury. Longitudinal studies tracking metabolic trajectories over time are therefore needed to identify biomarkers predictive of disease progression from steatosis to MASH, fibrosis, and cirrhosis. Second, although circulating metabolite signatures may correlate with liver pathology, they may not fully capture intrahepatic metabolic heterogeneity. Tissue-specific metabolic processes occurring within hepatocytes, immune cells, and stromal compartments may therefore remain partially obscured when analyses rely exclusively on plasma or serum samples. Integration of tissue metabolomics with imaging and spatial multi-omics approaches will likely provide deeper mechanistic insight into the metabolic architecture of MASLD. Finally, the translation of metabolomics discoveries into routine clinical practice requires robust validation across diverse populations, standardized analytical workflows, and cost-effective assays compatible with clinical laboratories. Bridging the gap between experimental metabolomics and clinical diagnostics will require coordinated efforts involving multicenter cohort studies, standardized reporting frameworks, and integration with established non-invasive diagnostic tools. Overall, while metabolomics has already transformed our understanding of MASLD pathophysiology, its greatest impact may emerge from integration with complementary omics technologies, standardized analytical frameworks, and large-scale longitudinal studies capable of defining reproducible metabolic endotypes across the spectrum of metabolic liver disease.

## 9. Controversial and Inconsistent Findings, Knowledge Gaps, and Future Research Priorities

Although metabolomic and lipidomic studies have substantially advanced the understanding of MASLD biology, several findings remain controversial or incompletely reconciled across cohorts. The most consistent signals involve lipid remodeling, bile acid disruption, amino acid imbalance, mitochondrial stress, and gut-derived metabolites. However, the specific metabolites, direction of change, and diagnostic performance of proposed panels vary considerably among studies. For example, some reports emphasize depletion of protective phosphatidylcholines, lysophosphatidylcholines, and polyunsaturated fatty acids during progression to MASH, whereas others identify stage-specific increases in selected lysophospholipids or acylcarnitines. Similarly, branched-chain and aromatic amino acids, bile acids, microbial co-metabolites and cholesterol-related metabolites may associate with steatosis, inflammation, fibrosis, or treatment response, but their relative importance differs depending on the population, biological matrix, and endpoint studied [134,135,136,137,138,139,140,141,142,143,144,145].

Several explanations may account for these discrepancies. First, MASLD is biologically heterogeneous: patients differ by obesity phenotype, T2D, insulin resistance, age, sex, menopausal status, ethnicity, diet, alcohol exposure, physical activity, medications, renal function, microbiome composition, and genetic background. These variables can alter circulating metabolites independently of liver histology and may obscure disease-specific signals if cohorts are insufficiently stratified. Second, studies often use different diagnostic anchors. A signature trained to identify advanced fibrosis may not perform equally well for active steatohepatitis, hepatocellular ballooning, early fibrogenesis, or future liver-related outcomes. Third, pre-analytical and analytical variability remains substantial. Fasting status, sample handling, storage time, extraction protocol, chromatography, ionization mode, batch correction, metabolite annotation confidence, and statistical modeling can all influence the metabolites detected and their reported direction of association [33,149,152,153,154,155,156].

A further source of inconsistency is the difference between circulating and intrahepatic biology. Plasma or serum profiles provide clinically attractive, minimally invasive readouts, but they integrate signals from the liver, adipose tissue, intestine, muscle, kidney, and immune cells. Consequently, a circulating biomarker may reflect systemic insulin resistance or gut microbial metabolism rather than a liver-specific pathogenic process. Conversely, liver tissue metabolomics captures local disease mechanisms but is constrained by biopsy sampling variability, regional heterogeneity, and limited feasibility in longitudinal studies. Spatial multi-omics partly addresses this limitation by mapping metabolic perturbations to specific hepatic niches, but these methods remain technically demanding and are not yet standardized for large clinical cohorts [23,33,155].

These uncertainties have important implications for future research priorities. The field should move beyond single-center discovery studies toward prospective, multicenter, longitudinal designs with harmonized sample collection, transparent reporting of metabolite identification confidence, shared reference materials and external validation across ancestries and care settings. Future studies should explicitly define the intended clinical use of each metabolomic model, from screening, fibrosis risk stratification, diagnosis of at-risk MASH, prediction of progression, and therapeutic monitoring to trial enrichment, rather than assuming transferability across endpoints. Machine-learning models require calibration, interpretability and comparison against established algorithms such as FIB-4, elastography, and imaging-based tools. Importantly, outcome-linked studies are needed to determine whether metabolomics-guided decisions improve referral efficiency, reduce unnecessary biopsy, identify treatment-eligible patients earlier, or predict liver-related events.

Remaining knowledge gaps also concern causality. Many metabolites associated with MASLD progression may be consequences rather than drivers of hepatocellular injury. Experimental perturbation, isotope tracing, organoid systems, human tissue validation and integration with genetics, transcriptomics, proteomics, microbiome profiling, and spatial biology will be required to distinguish biomarkers from mechanistic mediators. Attention should be given to sex- and reproductive-status-specific pathways, microbiome-derived metabolites, bile acid signaling, mitochondrial quality control, lipid-droplet cholesterol, and fibrogenic niche metabolism. Ultimately, resolving inconsistent findings will require a framework that treats MASLD not as a single metabolic state, but as a family of overlapping endotypes. Defining these endotypes reproducibly may allow metabolomics to evolve from descriptive profiling into clinically actionable precision hepatology.

Future studies should therefore classify metabolomic findings according to evidence level. At one end are exploratory disease-associated features identified by untargeted profiling; at the other are metabolites with experimental perturbation data, genetic support, reproducible tissue localization, or demonstrated effects on hepatocyte injury, mitochondrial function, immune activation, or stellate-cell fibrogenesis. Intermediate evidence includes replicated human associations, longitudinal prediction, pathway coherence, and consistency across biological matrices. Such evidence grading would help prevent overinterpretation of observational biomarkers while highlighting metabolites most suitable for therapeutic targeting.

To clarify the distinction between disease-associated biomarkers and metabolites with stronger mechanistic support, Table 5 summarizes selected metabolite classes according to the current type of evidence. This distinction is important because the same metabolite may have different meanings depending on biological matrix, disease stage, and study design. For example, a circulating metabolite may serve as a robust diagnostic marker without being a causal mediator, whereas a lipid species such as ceramide or DAG may function both as a measurable biomarker and as a participant in lipotoxic signaling. Future studies should therefore combine prospective human cohorts with mechanistic perturbation, isotope tracing, genetic analyses, organoid systems, and spatially resolved tissue validation to distinguish correlates, consequences, compensatory responses, and causal mediators.

## 10. Conclusions

Metabolomic and lipidomic investigations have begun to define convergent molecular signatures across the MASLD spectrum, linking circulating diagnostic panels, stage-dependent lipid remodeling, lipotoxic stress pathways and gut–liver axis-derived microbial metabolites. Recent multi-cohort analyses have refined this framework by identifying recurrent metabolomic fingerprints of MASLD across altered energy metabolism, essential and non-essential amino acids, fatty acids and bile acids. In parallel, serum metabolite-based diagnostic panels, including metabolites such as guanidinoacetate and saccharopine, have shown performance beyond conventional non-invasive indices for detecting significant fibrosis and inflammation [16]. The hepatic and circulating lipidomes have emerged as particularly informative readouts of disease progression. Across studies, the transition from steatosis to MASH is accompanied by stepwise depletion of protective PUFAs, LPCs and PCs, together with enrichment of DAGs and ceramides. These lipid species are not only biomarkers of metabolic derangement, but also plausible mediators of hepatic lipotoxicity, insulin resistance and inflammatory amplification [16,17,18,19,20].

Gut microbial metabolism provides an additional layer of disease biology. Lower levels of microbial metabolites such as indole propionic acid (IPA), an essential amino acid byproduct, have been associated with greater fibrosis severity and hepatocellular ballooning, supporting the concept that microbial–host co-metabolism contributes to inflammatory and fibrogenic progression in MASLD [19,21].

Spatially resolved metabolomics and transcriptomics now extend these observations from bulk molecular profiles to tissue architecture. By interrogating human liver tissue directly, integrative multi-omics studies reveal how macrophages and endothelial cells contribute to the metabolic microenvironment of advanced MASH. This spatial perspective suggests that disease-associated metabolic reprogramming can be mapped at cellular resolution, creating opportunities for more precise mechanistic stratification and spatially targeted therapeutic intervention [19,22,23,24]. Before routine clinical adoption, metabolomic biomarkers must therefore progress through analytical standardization, MSI-level annotation transparency, multicenter external validation, health-economic evaluation, and regulatory qualification for clearly defined clinical use cases.

Collectively, these studies position metabolomics as a bridge between mechanism and clinical translation in MASLD. The next challenge is to move from associative signatures to standardized, longitudinally validated and biologically interpretable biomarkers that can support risk prediction, therapeutic monitoring and precision trial design. Importantly, future precision-hepatology applications should distinguish metabolomic biomarkers that mark disease presence or stage from metabolites that are experimentally validated mediators of lipotoxicity, inflammation, mitochondrial dysfunction, or fibrogenesis.

## Figures and Tables

**Table 1:** Comparative overview of major metabolomic and multi-omic platforms used in MASLD research and clinical translation.

Platform	Main Advantages	Main Limitations	Clinical Applicability in MASLD
LC-MS	High sensitivity and broad metabolite coverage, especially for lipids, bile acids, acylcarnitines, amino acids, and polar metabolites; suitable for both untargeted discovery and targeted quantitative panels.	Requires careful sample preparation, chromatographic optimization, batch correction and metabolite annotation; inter-laboratory variability and limited standardization can affect reproducibility.	Currently the most versatile platform for MASLD biomarker discovery and targeted risk-stratification panels, particularly for MASH, fibrosis-related signatures, and treatment-response monitoring.
GC-MS	Robust chromatographic separation, strong compound-library matching, and high reproducibility for volatile or derivatized metabolites, including organic acids, sugars, short-chain fatty acids, and selected amino acids.	Limited to volatile or derivatizable compounds; derivatization increases preparation time and may introduce variability; less suitable for intact complex lipids than LC-MS.	Useful for focused analysis of energy metabolism, microbial co-metabolites, and selected small molecules in MASLD, but generally complementary rather than comprehensive for clinical biomarker panels.
NMR	Highly reproducible, non-destructive, quantitative, and minimally dependent on sample preparation; well suited for standardized high-throughput profiling of lipoproteins, amino acids, ketone bodies, and inflammatory glycoprotein signals.	Lower analytical sensitivity than MS-based methods and reduced coverage of low-abundance metabolites; spectral overlap may limit metabolite resolution.	Attractive for population studies, longitudinal cohorts and clinically scalable risk prediction, especially when reproducibility and cross-cohort comparability are priorities.
Spatial metabolomics	Preserves tissue context and maps metabolic alterations to hepatic zones, fibrotic niches, inflammatory foci, and cellular neighborhoods; can reveal regional disease mechanisms that are masked in bulk biofluids or tissue extracts.	Technically demanding, costly, and less standardized; spatial resolution, metabolite identification confidence, tissue preparation, and data integration remain major challenges.	Primarily a research platform at present, with strong value for understanding MASLD heterogeneity, liver zonation, fibrogenic microenvironments, and therapeutic targets, but limited immediate routine clinical use.
Multi-omics platforms	Integrate metabolomics with genomics, transcriptomics, proteomics, lipidomics, microbiome data, imaging, and clinical variables to define disease endotypes and improve biological interpretation.	Require large, well-phenotyped cohorts, advanced bioinformatics, harmonized pipelines, and external validation; the risk of overfitting and limited interpretability remains substantial.	Most promising for precision medicine, patient stratification, trial enrichment, and mechanism-informed biomarker development, but clinical deployment requires validated algorithms, cost-effectiveness evidence and workflow integration.

**Table 2:** Molecular and metabolic pathways associated with progression to MASH.

Pathophysiological Axis	Primary Markers or Disruption Points	Functional Consequence in MASLD–MASH Progression	Ref.
Lipotoxicity	Palmitate, ceramides, DAGs, saturated fatty acids, free cholesterol, CD36 upregulation and altered phospholipid remodeling.	Promotes insulin resistance, ER stress, mitochondrial injury, hepatocyte apoptosis or lytic cell death, and the transition from adaptive lipid storage to lipotoxic MASH.	[37,44,45,51]
Mitochondrial dysfunction	Reduced oxidative phosphorylation through complexes I, II and IV, altered β-oxidation, citrate and succinate overflow, megamitochondria, loss of membrane potential and cristae disorganization.	Converts compensatory lipid oxidation into inefficient energy metabolism, oxidative stress and inflammatory metabolic signaling, thereby supporting progression from steatosis to MASH.	[53,56,59,60]
Oxidative and organelle stress	Malondialdehyde, 3-nitrotyrosine, mitochondrial reactive oxygen species, oxidized mitochondrial DNA, ER stress, membrane lipid peroxidation and permeability transition.	Amplifies hepatocyte ballooning, membrane damage and release of damage-associated molecular patterns, linking metabolic overload to sterile inflammatory activation.	[61,63,66,67]
Inflammatory signaling	NLRP3 inflammasome activation, cGAS–STING and TLR9 signaling, NF-κB-JNK activation, IL-1β, IL-18, TNF-α, IL-6, IL-8 and CCL2/MCP-1.	Transforms lipotoxic injury into a self-amplifying inflammatory program that recruits Kupffer cells, monocyte-derived macrophages, neutrophils and lymphoid cells.	[68,69,70,71]
Fibrogenic remodeling	Hepatic stellate-cell activation, TGF-β1–Smad signaling, PDGF–PDGFRβ signaling, metabolic rewiring, LSEC capillarization and extracellular-matrix deposition.	Links chronic inflammation to matrix production, sinusoidal remodeling, progressive fibrosis and the emergence of the MASH fibrotic niche.	[73,80,84,86,87]

Abbreviations used: CCL2, C-C motif chemokine ligand 2; CD36, cluster of differentiation 36; DAGs, diacylglycerols; ER, endoplasmic reticulum; IL-1β, interleukin-1 beta; IL-6, interleukin-6; IL-8, interleukin-8; IL-18, interleukin-18; JNK, c-Jun N-terminal kinase; LSEC—liver sinusoidal endothelial cell; MCP-1, monocyte chemoattractant protein-1; NF-κB, nuclear factor kappa-light-chain-enhancer of activated B cells; NLRP3, NOD-, LRR- and pyrin domain-containing protein 3; PDGF, platelet-derived growth factor; PDGFRβ, platelet-derived growth factor receptor beta; Smad, suppressor of mothers against decapentaplegic; TGF-β1, transforming growth factor beta 1; TNF-α, tumor necrosis factor alpha.

**Table 3:** Metabolomics-based models for non-invasive risk stratification in MASLD and MASH.

Author/Year/Ref	Method	Findings	Conclusions
Caussy et al., 2019 [134]	Cross-sectional derivation cohort of biopsy-proven NAFLD plus two validation cohorts; untargeted serum metabolomics and comparison with FIB-4 and NAFLD Fibrosis Score.	Thirty-two of 652 metabolites were associated with advanced fibrosis. A 10-metabolite panel showed high diagnostic performance for advanced fibrosis, with AUROC 0.94 in derivation and strong performance in validation cohorts.	A serum metabolite panel may provide a non-invasive blood-based tool for detecting advanced fibrosis with better accuracy than conventional fibrosis scores.
Piras et al., 2021 [135]	Systematic review following PRISMA guidelines of metabolomics studies in NAFLD/NASH published from 2010 to 2021.	Ten eligible studies were identified. Amino acid, fatty acid, and vitamin metabolism differed between NAFLD/NASH patients and controls, with overlap between NAFLD/NASH and metabolic syndrome.	Low-molecular-weight metabolites are promising indicators of NAFLD/NASH and may support early diagnosis, biomarker discovery, and therapeutic target identification.
Shoji et al., 2022 [136]	Targeted LC-MS/MS analysis of bile acids and sterols in a mouse NASH model induced by a choline-deficient, methionine-reduced high-fat diet.	Before NASH onset, desmosterol, 4β-hydroxycholesterol, campesterol, sitosterol, and secondary bile acids decreased, while autoxidation-generated sterols and primary bile acids increased.	Early changes in cholesterol metabolites may reflect inflammation and detoxification processes and could help identify early diagnostic biomarkers for NASH.
Ji et al., 2022 [137]	Targeted plasma metabolomics using GC-MS/MS and LC-MS/MS, machine-learning analyses, and integration with liver RNA-seq data in healthy controls, NAFL, and NASH patients.	Six metabolites differed significantly in NAFLD. Machine-learning models identified eight discriminating metabolites, and three metabolites—glutamic acid, isocitric acid, and aspartic acid—formed the MetaNASH score.	The MetaNASH score showed strong potential for distinguishing NASH and for linking circulating metabolites with liver metabolic pathway alterations.
Ahmed et al., 2022 [138]	Targeted and non-targeted metabolomics plus isotope-labeled lipidomics comparing NASH and NASH-HCC patients.	NASH-HCC patients showed higher triacylglycerol, AFP, AST, and CA19-9, and a 20-metabolite panel separated NASH-HCC from NASH. Altered pathways included lipid metabolism, bile acids, DNA repair, methylation, necroptosis, and mTOR signaling.	Distinct metabolite fingerprints may reveal metabolic reprogramming involved in progression from NASH to HCC.
Ganesan et al., 2023 [139]	Untargeted stool metabolomics and clinical data analysis in healthy controls, NAFL, NASH, and cirrhosis patients using GC-MS and LC-TOF-MS.	One hundred three metabolites were identified. Short-chain fatty acids were reduced in NAFLD, stool cholic acid increased, and palmitoylcarnitine and L-carnitine increased in NASH and cirrhosis.	NAFLD stages show distinct gut-related metabolomic profiles, supporting microbial metabolites as potential indicators of gut–liver metabolic severity.
Dorochow et al., 2023 [140]	Mouse models of alcoholic and non-alcoholic steatohepatitis analyzed by liver lipidomics, metabolomics, and immune-cell profiling.	NASH showed greater lipid droplet size, oxidative stress, uremic metabolites, tryptophan/kynurenine changes, and altered macrophage recruitment and polarization compared with ASH.	Despite comparable disease severity, NASH and ASH show disease-specific metabolic and immunological signatures that may reflect distinct pathomechanisms.
Zhang et al., 2023 [141]	Integrated plasma metabolomics and lipidomics with liver proteomics in NASH patients, plus evaluation of cholestyramine in a NASH mouse model.	NASH was characterized by altered fatty-acid uptake, lipid droplet proteins, glycolysis, branched-chain and aromatic amino acids, purines, and bile acids. Cholestyramine reduced steatosis, fibrosis, bile acids, and steroid hormones in mice.	Multi-omics profiling defines key metabolic disturbances in NASH and suggests bile acid modulation as a therapeutic strategy.
Noureddin et al., 2024 [142]	Derivation and validation cohorts from international tertiary centers; liver biopsy, laboratory data, and metabolomics-driven MASEF score based on 12 lipids, BMI, AST, and ALT.	MASEF identified at-risk MASH with AUROC 0.76 in derivation and 0.79 in validation. FIB-4 plus MASEF performed similarly to FIB-4 plus liver stiffness by VCTE.	MASEF is a promising non-invasive tool for identifying at-risk MASH and may be an alternative to liver stiffness measurement in diagnostic algorithms.
Ogiso et al., 2024 [143]	Integrated non-targeted and targeted metabolomics in hepatocyte-specific PTEN knockout mice with MASH, with and without apomorphine treatment.	LPLs were elevated in MASH and decreased after apomorphine treatment, indicating modulation of disease-related metabolic disruption.	LPLs may be central to MASH pathogenesis and potential therapeutic targets; apomorphine showed potential to modify MASH-associated metabolic alterations.
Huang et al., 2025 [144]	Machine-learning development of a MASH prediction score using 44 clinical parameters and 250 serum metabolites in a Chinese biopsy cohort; external Finnish validation and mortality analysis in population cohorts.	The final score included BMI, AST, tyrosine, and VLDL phospholipid-to-total lipid ratio. It predicted MASH with AUROC 0.87 in Chinese and 0.81 in Finnish cohorts and was associated with MASLD-related mortality.	A metabolome-derived score can predict MASH and liver-related mortality across Chinese and European populations, outperforming established fibrosis scores for mortality prediction.
Wu et al., 2026 [145]	Deep multimodal fusion framework integrating SERS spectra, clinical-biochemical parameters, and bile acid metabolomics in a biopsy-proven MASLD cohort.	MedFusionNet outperformed conventional and multimodal baseline models for non-invasive MASH diagnosis and provided interpretable modality-level contributions through SHAP analysis.	Multimodal deep learning may provide a scalable and clinically actionable framework for early, non-invasive MASH detection.

Abbreviations used: AFP—alpha-fetoprotein; ALT—alanine aminotransferase; AST—aspartate aminotransferase; ASH—alcohol-related steatohepatitis; AUROC—area under the receiver operating characteristic curve; BMI—body mass index; CA19-9—carbohydrate antigen 19-9; FIB-4—fibrosis-4 index; GC-MS—gas chromatography–mass spectrometry; GC-MS/MS—gas chromatography–tandem mass spectrometry; HCC—hepatocellular carcinoma; LC-MS/MS—liquid chromatography–tandem mass spectrometry; LC-TOF-MS—liquid chromatography–time-of-flight mass spectrometry; LPLs—lysophospholipids; MASEF- metabolomics-advanced steatohepatitis fibrosis score; MASH—metabolic dysfunction-associated steatohepatitis; NAFL—nonalcoholic fatty liver; NAFLD—nonalcoholic fatty liver disease; NASH—nonalcoholic steatohepatitis; PTEN—phosphatase and tensin homolog; RNA-seq—RNA sequencing; SERS—surface-enhanced Raman spectroscopy; SHAP—SHapley Additive exPlanations; VCTE—vibration-controlled transient elastography; VLDL—very-low-density lipoprotein.

**Table 4:** Key barriers and requirements for clinical implementation of metabolomic, spatial, single-cell, AI-assisted, and multimodal biomarkers in MASLD.

Barrier	Why It Matters	Required Action Before Routine Clinical Use
Inter-platform variability	LC-MS, GC-MS, NMR, spatial metabolomics, and multi-omics platforms differ in metabolite coverage, sensitivity, dynamic range, quantitative accuracy, spatial resolution, and reproducibility.	Perform platform-specific validation, inter-laboratory comparison, bridging studies, harmonized assay protocols, and use of reference materials.
Batch effects and pre-analytical variation	Instrument drift, sample order, reagent lots, operator differences, matrix effects, fasting status, processing time, storage temperature, freeze–thaw cycles, tissue ischemia time, embedding method, and long-term storage conditions can confound biological signals.	Use pooled quality-control samples, internal standards, batch randomization, drift correction, normalization procedures, standardized sample-handling protocols, and prespecified quality-control thresholds.
Metabolite annotation confidence	Putative annotations may lead to incorrect biological interpretation, non-transferable biomarkers, or regulatory uncertainty.	Report annotation confidence according to Metabolomics Standards Initiative levels and prioritize MSI level 1 confirmation for clinically intended biomarkers using authentic standards, matched retention time, and fragmentation spectra.
Spatial and single-cell metabolomics	Spatial and single-cell approaches preserve tissue context and cell-type information, but face challenges related to tissue handling, spatial resolution, ion suppression, low metabolite abundance, throughput, metabolite annotation, and integration with transcriptomic or imaging data.	Standardize tissue preparation, spatial resolution reporting, metabolite annotation confidence, imaging and omics integration pipelines, and validate spatial signatures in independent tissue cohorts.
Pipeline standardization	Differences in peak detection, feature alignment, normalization, missing-data handling, batch correction, metabolite annotation, image registration, spatial segmentation, and statistical or machine-learning modeling reduce reproducibility.	Develop locked analytical and computational workflows with documented preprocessing steps, predefined modeling strategies, transparent reporting, and quality-management procedures.
AI-assisted and multimodal model integration	Machine-learning and deep-learning models may combine metabolomics with clinical data, imaging, spectroscopy, genetics, microbiome, elastography, or spatial omics, but can be affected by overfitting, data leakage, poor calibration, limited interpretability, site-specific bias, and dataset shift.	Use locked algorithms, transparent model reporting, SHAP or related interpretability tools, calibration analyses, external validation, prospective clinical testing, and direct comparison with established non-invasive pathways.
External validation	Models derived from tertiary-care, biopsy-enriched, or geographically restricted cohorts may overestimate diagnostic accuracy in real-world settings.	Validate independently in multicenter cohorts across primary-care, specialist, and community populations.
Population diversity	Diet, ethnicity, ancestry, sex, menopausal status, age, obesity phenotype, type 2 diabetes, medications, renal function, alcohol exposure, genetics, and microbiome composition influence metabolite profiles.	Test performance across diverse demographic, metabolic, genetic, and geographic groups and define population-specific thresholds when required.
Clinical utility	Diagnostic discrimination alone does not prove that a test improves patient management or outcomes.	Conduct prospective implementation studies assessing referral efficiency, biopsy avoidance, treatment eligibility, monitoring performance, liver-related outcomes, and comparison with FIB-4, elastography, imaging, and established blood tests.
Cost-effectiveness and workflow integration	High assay costs, long turnaround times, limited laboratory availability, uncertain reimbursement, computational burden, and poor integration into clinical workflows may limit scalability.	Perform health-economic analyses, define reimbursement strategies, ensure clinically useful turnaround times, and integrate results into electronic health records and decision pathways.
Regulatory qualification	Tests used for diagnosis, treatment selection, trial enrichment, or monitoring require formal validation and oversight, especially when based on AI-assisted or multimodal algorithms.	Define intended use, analytical performance specifications, clinical validation requirements, locked algorithms, laboratory certification, quality-management procedures, and post-implementation performance monitoring.

Abbreviations used: AI, artificial intelligence; FIB-4, fibrosis-4 index; GC-MS, gas chromatography–mass spectrometry; LC-MS, liquid chromatography–mass spectrometry; MASLD, metabolic dysfunction-associated steatotic liver disease; MSI, Metabolomics Standards Initiative; NMR, nuclear magnetic resonance; SHAP, SHapley Additive exPlanations.

**Table 5:** Disease-associated versus mechanistically supported metabolomic signals in MASLD.

Metabolite or Pathway Class	Main Association in MASLD/MASH	Current Evidence Type	Interpretation
Multi-metabolite diagnostic panels	Identify steatosis, at-risk MASH, advanced fibrosis, or MASH-related HCC in selected cohorts.	Mostly observational, cross-sectional, or machine-learning-derived; some external validation.	Useful as biomarkers for risk stratification, but individual metabolites within panels should not be assumed to be causal mediators without experimental validation.
Diacylglycerols	Accumulate during lipid overload and associate with insulin resistance and progression from steatosis to MASH.	Human lipidomics plus mechanistic cellular and experimental evidence linking DAGs to PKC activation.	Biomarker and plausible mediator of hepatic insulin resistance.
Ceramides	Increased in lipotoxic states and associated with insulin resistance, mitochondrial dysfunction, oxidative stress, and hepatocyte apoptosis.	Human association studies plus experimental evidence showing disruption of Akt/PKB signaling, mitochondrial injury, ROS generation, and apoptosis.	Biomarker and mechanistically supported lipotoxic mediator.
Saturated fatty acids, especially palmitate	Associated with lipotoxic stress, ER stress, mitochondrial dysfunction, inflammatory signaling, and hepatocyte death.	Strong mechanistic evidence from cell and animal studies, supported by human metabolic context.	Mechanistically supported contributor to lipotoxic MASH.
Free cholesterol and lipid-droplet cholesterol	Enriched in MASH and fibrosis and linked to PNPLA3 and HSD17B13 genetic backgrounds.	Human tissue evidence plus animal-model and mechanistic data.	Mechanistically plausible mediator of fibro-inflammatory progression, not merely a passive biomarker.
Phosphatidylcholines, lysophosphatidylcholines, and PUFAs	Depletion often associates with transition from steatosis to MASH and altered membrane integrity.	Predominantly lipidomic association studies, with mechanistic plausibility from membrane biology and ER stress pathways.	Primarily biomarkers of lipid remodeling, with plausible but context-dependent mechanistic relevance.
Acylcarnitines and TCA-cycle intermediates	Associated with incomplete fatty-acid oxidation, mitochondrial stress, and altered energy metabolism.	Mostly observational in humans; mechanistic support from mitochondrial biology and experimental models.	Biomarkers of mitochondrial adaptation or dysfunction; causal contribution requires flux-based confirmation.
Bile acids	Altered bile acid pools associate with steatosis, inflammation, fibrosis, cholestasis, and gut–liver axis dysregulation.	Human association studies plus mechanistic evidence through FXR/TGR5 signaling and experimental bile-acid modulation.	Both biomarkers and signaling mediators, depending on bile acid species and disease context.
Microbial metabolites, including SCFAs, indole derivatives, TMA/TMAO, ethanol/acetaldehyde, and lipopolysaccharide	Associate with gut dysbiosis, intestinal permeability, inflammation, fibrosis, and cardiometabolic risk.	Mixed evidence; strong mechanistic plausibility and experimental support for selected metabolites, but many human MASLD data remain associative.	Biomarkers of gut–liver–adipose crosstalk; selected metabolites may act as mechanistic mediators.
Amino acids, including BCAAs and aromatic amino acids	Associate with insulin resistance, advanced fibrosis, cirrhosis, and altered Fischer’s ratio.	Largely observational in MASLD; strong clinical association in cirrhosis and systemic metabolic disease.	Useful staging or systemic metabolic biomarkers; causal role in MASLD progression remains incompletely defined.
Mitochondrial damage-associated metabolites and oxidized mtDNA	Associated with oxidative stress, inflammatory activation, ballooning, and MASH progression.	Mechanistic evidence from mitochondrial injury, cGAS-STING, TLR9, and inflammasome studies.	Mechanistically supported injury signals, although circulating measures require careful interpretation.

Abbreviations used: BCAA, branched-chain amino acid; DAG, diacylglycerol; ER, endoplasmic reticulum; FXR, farnesoid X receptor; HCC, hepatocellular carcinoma; HSD17B13, hydroxysteroid 17-beta dehydrogenase 13; MASLD, metabolic dysfunction-associated steatotic liver disease; MASH, metabolic dysfunction-associated steatohepatitis; mtDNA, mitochondrial DNA; PKC, protein kinase C; PNPLA3, patatin-like phospholipase domain-containing 3; PUFA, polyunsaturated fatty acid; ROS, reactive oxygen species; SCFA, short-chain fatty acid; TCA, tricarboxylic acid; TGR5, Takeda G protein-coupled receptor 5; TLR9, toll-like receptor 9; TMA, trimethylamine; TMAO, trimethylamine N-oxide.

## Data Availability

No new data were created or analyzed in this study. Data sharing is not applicable to this article.

## Abbreviations

The following abbreviations are used in this manuscript:

ACC	acetyl-CoA carboxylase
ADMA	asymmetric dimethylarginine
AFP	alpha-fetoprotein
ALT	alanine aminotransferase
ASH	alcohol-related steatohepatitis
AST	aspartate aminotransferase
ATGL	adipose triglyceride lipase
AUROC	area under the receiver operating characteristic curve
BCAAs	branched-chain amino acids
BMI	body mass index
CA19-9	carbohydrate antigen 19-9
CCL2	C-C motif chemokine ligand 2
CD36	cluster of differentiation 36
CHOP	C/EBP homologous protein
DCA	deoxycholic acid
DGAT	diacylglycerol acyltransferase
DNL	de novo lipogenesis
ER	endoplasmic reticulum
ERK	extracellular signal-regulated kinase
FASN	fatty acid synthase
FIB-4	fibrosis-4 index
FFAs	free fatty acids
FXR	farnesoid X receptor
GC-MS	gas chromatography–mass spectrometry
GC-MS/MS	gas chromatography–tandem mass spectrometry
GCKR	glucokinase regulator
GPCRs	G-protein–coupled receptors
GPR91	G-protein–coupled receptor 91
HCC	hepatocellular carcinoma
HSC(s)	hepatic stellate cell(s)
HSD17B13	hydroxysteroid 17-beta dehydrogenase 13
IL-1β	interleukin-1 beta
IL-6	interleukin-6
IL-8	interleukin-8
IL-18	interleukin-18
IPA	indole propionic acid
JNK	c-Jun N-terminal kinase
LC-MS	liquid chromatography–mass spectrometry
LC-MS/MS	liquid chromatography–tandem mass spectrometry
LC-TOF-MS	liquid chromatography–time-of-flight mass spectrometry
LPC(s)	lysophosphatidylcholine(s)
LPLs	lysophospholipids
LSEC(s)	liver sinusoidal endothelial cell(s)
MAFLD	metabolic dysfunction-associated fatty liver disease
MASEF	metabolomics-advanced steatohepatitis fibrosis score
MASH	metabolic dysfunction-associated steatohepatitis
MASLD	metabolic dysfunction-associated steatotic liver disease
MBOAT7	membrane-bound O-acyltransferase domain-containing 7
MCP-1	monocyte chemoattractant protein-1
MDA	malondialdehyde
MFN1	mitofusin 1
MFN2	mitofusin 2
MSI	Metabolomics Standards Initiative
mTOR	mechanistic target of rapamycin
mtDNA	mitochondrial DNA
NAFL	nonalcoholic fatty liver
NAFLD	nonalcoholic fatty liver disease
NASH	nonalcoholic steatohepatitis
NF-κB	nuclear factor kappa-light-chain-enhancer of activated B cells
NLRP3	NOD-, LRR- and pyrin domain-containing protein 3
NMR	nuclear magnetic resonance
OPA1	optic atrophy 1
PC(s)	phosphatidylcholine(s)
PMOS	Polyendocrine metabolic ovary syndrome
PDGF	platelet-derived growth factor
PDGFRβ	platelet-derived growth factor receptor beta
PE	phosphatidylethanolamine
PI3K	phosphoinositide 3-kinase
PINK1	PTEN-induced kinase 1
PKB	protein kinase B
PKC	protein kinase C
PNPLA3	patatin-like phospholipase domain-containing 3
PTEN	phosphatase and tensin homolog
PUFAs	polyunsaturated fatty acids
RNA-seq	RNA sequencing
SCFAs	short-chain fatty acids
SERS	surface-enhanced Raman spectroscopy
SHAP	SHapley Additive exPlanations
SLD	steatotic liver disease
Smad	suppressor of mothers against decapentaplegic
SREBP-1c	sterol regulatory element-binding protein 1c
TCA	tricarboxylic acid
TGF-β1	transforming growth factor beta 1
TLR4	toll-like receptor 4
TLR9	toll-like receptor 9
TMA	trimethylamine
TMAO	trimethylamine N-oxide
TM6SF2	transmembrane 6 superfamily member 2
TNF-α	tumor necrosis factor alpha
VCTE	vibration-controlled transient elastography
VLDL	very low-density lipoprotein
YAP	yes-associated protein
